# Human Cytomegalovirus Infection Elicits New Decidual Natural Killer Cell Effector Functions

**DOI:** 10.1371/journal.ppat.1003257

**Published:** 2013-04-04

**Authors:** Johan Siewiera, Hicham El Costa, Julie Tabiasco, Alain Berrebi, Géraldine Cartron, Philippe Bouteiller, Nabila Jabrane-Ferrat

**Affiliations:** 1 Institut National de la Santé et de la Recherche Médicale; UMR 1043, Toulouse, France; 2 Centre National Recherche Scientifique; UMR 5282, Toulouse, France; 3 Université Toulouse III Paul Sabatier, Toulouse, France; 4 Service de Gynécologie-Obstétrique, Centre Hospitalo-Universitaire de Toulouse, Hôpital Paule de Viguier, Toulouse, France; University of Rijeka, Croatia

## Abstract

During the first trimester of pregnancy the uterus is massively infiltrated by decidual natural killer cells (dNK). These cells are not killers, but they rather provide a microenvironment that is propitious to healthy placentation. Human cytomegalovirus (HCMV) is the most common cause of intrauterine viral infections and a known cause of severe birth defects or fetal death. The rate of HCMV congenital infection is often low in the first trimester of pregnancy. The mechanisms controlling HCMV spreading during pregnancy are not yet fully revealed, but evidence indicating that the innate immune system plays a role in controlling HCMV infection in healthy adults exists. In this study, we investigated whether dNK cells could be involved in controlling viral spreading and in protecting the fetus against congenital HCMV infection. We found that freshly isolated dNK cells acquire major functional and phenotypic changes when they are exposed to HCMV-infected decidual autologous fibroblasts. Functional studies revealed that dNK cells, which are mainly cytokines and chemokines producers during normal pregnancy, become cytotoxic effectors upon their exposure to HCMV-infected autologous decidual fibroblasts. Both the NKG2D and the CD94/NKG2C or 2E activating receptors are involved in the acquired cytotoxic function. Moreover, we demonstrate that CD56^pos^ dNK cells are able to infiltrate HCMV-infected trophoblast organ culture *ex-vivo* and to co-localize with infected cells *in situ* in HCMV-infected placenta. Taken together, our results present the first evidence suggesting the involvement of dNK cells in controlling HCMV intrauterine infection and provide insights into the mechanisms through which these cells may operate to limit the spreading of viral infection to fetal tissues.

## Author Summary

Human cytomegalovirus (HCMV) is a herpes virus that can establish persisting infection in immunocompetent hosts. HCMV primary infection during pregnancy is devastating; it can result in up to 75% of congenital infections and it is a known cause of fetal death. The immune system and particularly natural killer cells (NK) are known to play a key role in the clearance of several viruses in healthy adults. Whether decidual NK cells (dNK), present in the pregnant uterus, have a role during HCMV infection is not known. We analyze changes in dNK cell function and phenotype in the presence of HCMV-infected targets in an autologous setting. We demonstrate the acquisition of cytotoxic profile which is associated with changes in dNK cell receptor repertoire and cytokine production. Finally, we find that dNK cells are able to sense HCMV infection, migrate and infiltrate infected tissues both in tissular organ culture and *in situ* in infected placenta. Together our results present the first report demonstrating the involvement of dNK cells in controlling HCMV infection.

## Introduction

Human cytomegalovirus (HCMV) infection is mostly asymptomatic in healthy adults and results in the establishment of long term latency. On the contrary, life threatening diseases may occur in immunocompromised patients after viral reactivation or primary HCMV infections. HCMV is the most common cause of intra-uterine viral infections and a leading cause of congenital infection [1], [2]. Even though maternal-fetal transmission is not systematic [3], the prevalence of HCMV transmission is about 30% in the first trimester of pregnancy and can reach up to 72% in the third trimester [4]. It is believed that the first steps of infection and amplification take place in the *decidua* where both maternal and fetal cells are in close contact [5].

Human placentation is associated with a large increase of decidual NK cells (dNK). During the first trimester of pregnancy, dNK cells are the major population of maternal immune cells as they count for 70% of total immune cells present in the *decidua* in the first trimester of pregnancy [6], [7], whereas other immune cells, macrophages, T cells (including CD8, CD4 and γδ T cells) and dendritic cells count for 20, 10 and 2% respectively. The role of dNK cells during pregnancy is not yet fully understood. Their contribution to successful placentation versus their potential ability to exert cytotoxicity remains a major paradox [8], [9]. By secreting a unique profile of cytokines/chemokines and angiogenic factors, dNK cells are thought to be crucial for successful placentation and materno-fetal immune tolerance [9]–[15]. dNK cells exhibit different phenotypic and functional characteristics from other peripheral blood NK cells (pNK). The majority of dNK cells are CD56^bright^CD16^neg^ and they express a repertoire of activating and inhibitory receptors (NKRs) that resembles that of early differentiation stages of pNK cells [9], [16]–[19]. The lack of dNK cell cytotoxicity has been attributed to defects in the formation of the immunological synapse and/or failure of 2B4 receptor to convey activating signals [8], [20], [21].

In contrast to the clearly defined role of human and mouse pNK cells in controlling viral infections [22]–[31], little is known about the ability of dNK cells to control viral infections during pregnancy [17], [32], [33]. dNK cells represent the major decidual lymphoid population in the first trimester of pregnancy [7], [6] and vertical transmission of HCMV to the fetus is quite low during this trimester, therefore it is conceivable that dNK cells might be involved in limiting HCMV viral spreading to fetal tissues. To test this possibility, we have conducted detailed analysis of functional and phenotypic changes of first trimester of pregnancy dNK cells after their exposure to infected target autologous fibroblasts. We found that dNK cells acquire cytotoxic effector function that is associated with phenotypic alterations in their receptor repertoire expression and involves key receptor-ligand pairs. Furthermore, we found that dNK cells were able to sense HCMV infection, migrate and infiltrate placental tissues both in tissue organ culture and *in situ* in HCMV-infected placenta. These results suggest that dNK cells control HCMV spreading across mucosal tissues probably through the acquisition of cytotoxic profile.

## Results

### Decidual NK cells efficiently kill HCMV-infected autologous target cells

Our previous study provided evidence indicating that cytolytic function of dNK cells during normal pregnancy is partially controlled by negative signals that involve NKG2A receptor [9] suggesting that such function might be modulated upon viral infections. Therefore, to test the possible involvement of dNK cells in controlling HCMV infection we examined their cytotoxic effector function against HCMV-infected autologous decidual fibroblasts. dNK cells and decidual fibroblasts (Figure S1A) were purified from the same *decidua basalis*. High purity fibroblasts (Vimentin^pos^ and Cytokeratin-7^neg^, Figure S1A) were infected with two strains of HCMV; the VHLE clinical isolate and the laboratory strain AD169. Decidual fibroblasts were efficiently infected by both strains as evidenced by staining for HCMV-IE nuclear protein (Figure S1B and data not shown) where more than 60%±3 (mean ± S.D.) of cells were infected after 48 h (Figure S1C).

**Figure 1:**
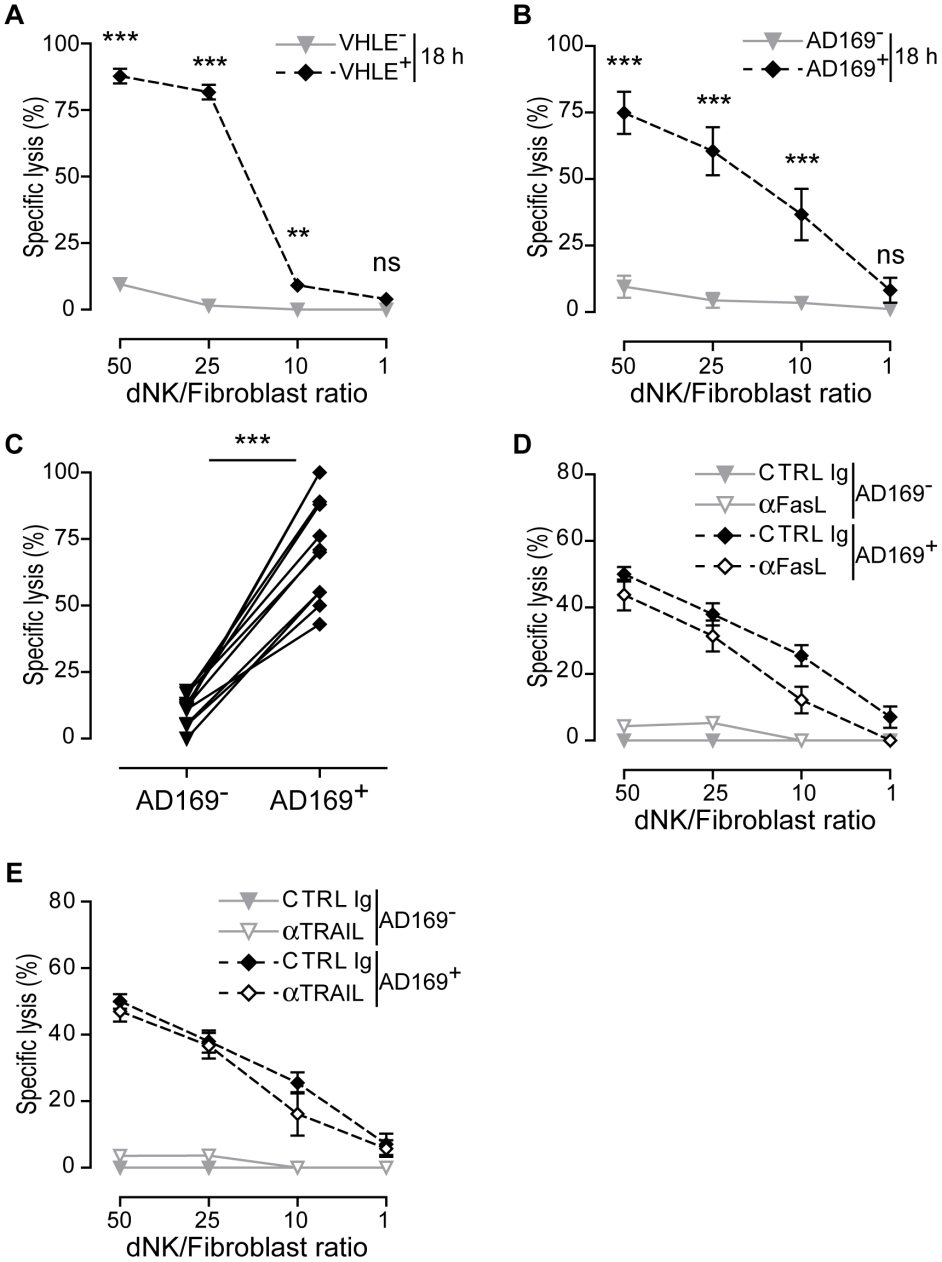
dNK cells are cytotoxic against HCMV-infected autologous fibroblasts. Decidual fibroblasts were kept uninfected or infected for 48 h with HCMV. dNK cell cytotoxicity was determined by ^51^Cr-release assay after 18 hours of contact at different E/T ratios. (A) Fibroblasts were infected with VHLE clinical isolate (n = 3), (B) cells infected with AD169 laboratory strain of HCMV (n = 5). (C) Analysis of dNK cell cytotoxicity from a cohort of 10 *decidua* samples at the 50 to 1 ratio. (D) dNK cells were pre-incubated with anti-FasL or (E) anti-TRAIL blocking mAbs at the final concentration of 10 µg/ml for 20 min and cytotoxicity was monitored after 18 h. Control (CTRL), lysis performed in the presence of IgG control. Each data point is calculated as the mean lysis ± S.D. from at least five independent experiments done in replicate tissue culture wells. Statistical comparisons of mean lysis of uninfected versus HCMV-infected were performed using two-way ANOVA test. ***, *p*<0.001; **, *p*<0.01; ns, not significant, *p*>0.05.

Through co-cultures in autologous settings, we then investigated the cytotoxicity of dNK cells by conventional chromium release assay. Neither dNK cells nor pNK cells killed efficiently HCMV-infected decidual fibroblasts after 4 h of contact (Figure S2A & B). However, after 18 h dNK cells efficiently killed VHLE- or AD169-infected fibroblasts (Figure 1A & B). With both strains up to 75% of killing was reached at the effector to target ratio of 50 and no killing of autologous uninfected fibroblasts was observed indicating the specificity of the cytotoxic function against HCMV-infected targets. Significant increases were also observed in pNK cell lysis of HCMV-infected autologous fibroblasts after 18 h of contact (Figure S2C). Given that no major differences were observed between VHLE or AD169 strains, we extended the analysis of dNK cell cytotoxicity to a cohort of 10 *decidua basalis* and confirmed that dNK cells can specifically kill AD169-infected fibroblasts, although with some variable efficiency (Figure 1C). Taken together, these data suggest that under HCMV infection dNK cells become cytotoxic against infected autologous fibroblasts.

To further confirm the cytotoxic function of dNK cells, we next investigated lytic capacities of dNK cells in an MHC mismatched (heterologous) setting (Figure S2D–E). dNK cells were purified from one *decidua basalis* and their killing activity was tested against either uninfected or HCMV-infected heterologous decidual fibroblasts. While very little killing was observed after 4 h of contact (Figure S2D), up to 60% of uninfected and HCMV-infected heterologous fibroblasts were killed after 18 h of contact (Figure S2E). To exclude any external bias that could be responsible for initiating dNK cell cytotoxicity against heterologous fibroblasts, we tested the ability of dNK cells to kill K562 classical NK cell targets (Figure S2F). In agreement with previous studies [21], very little lysis was observed in the presence of dNK cells while pNK cells killed up to 75% of K562 cells (Figure S2F). Further analyses demonstrate that while dNK cells killed more than 55% of HCMV-infected autologous fibroblasts after 18 h of contact, they did not kill semi-allogeneic fetal trophoblasts (Figure S2G). In the same manner pNK cells did not kill semi-allogeneic trophoblasts (data not shown).

These observations suggest that dNK cells that are tolerant both *in vivo* and *in vitro* to semi-allogeneic fetal trophoblasts become activated when there is a danger signal such as HCMV-infection.

### Cytotoxic effector function of dNK cells is independent of TRAIL or FasL killing pathway

NK cells achieve target cell killing either through delivery of soluble mediators or by triggering death receptor-ligand pathways such as Fas ligand (FasL) or the tumor necrosis factor-related apoptosis-inducing ligand (TRAIL). To provide insights into the mechanisms involved in dNK cell killing of HCMV-infected fibroblasts, we investigated the involvement of the death receptor-ligand pathway (Figure 1D–E). We used neutralizing antibodies to either TRAIL or FasL that are expressed on dNK cells, to block their interaction with cognate death receptors expressed on target cells. After 18 h of co-culture, the blockade of either FasL (Figure 1D) or TRAIL (Figure 1E) did not affect dNK cell cytotoxicity against HCMV-infected autologous fibroblasts. The blocking ability of both mAbs was confirmed since they prevented TRAIL- or FasL-induced killing of Jurkat cell line (see Figure S2H). These data strongly suggest that dNK cell killing of HCMV-infected fibroblasts proceeds through mechanisms independent of the death receptor-ligand pathways.

### dNK cells engage immune synapse with HCMV-infected autologous fibroblasts and polarize their lytic machinery towards HCMV-infected targets

The delivery of perforin/granzyme lethal hits is a highly regulated multistep mechanism that involves the formation of a dynamic structure, namely immunological synapse (IS), between NK cell and its target [34]. We undertook a stepwise approach to dissect the involvement of perforin-induced killing mechanisms. First, we analyzed the capacity of dNK cells to form IS with autologous targets. Conjugates formation between dNK cells and uninfected/HCMV-infected autologous decidual fibroblasts was analyzed after 20 min of interaction by monitoring F-actin remodeling and confocal microscopy. Although dNK cells recognized both uninfected and HCMV-infected target cells, as evidenced by their actin-enriched flattened shape (Figure S3A), only 17% uninfected cells were engaged in conjugates with dNK cells while more than 55% AD169-infected fibroblasts were recognized by dNK cells (Figure S3B). Thus, dNK cells form conjugates preferentially with HCMV-infected fibroblasts and reorganize their F-actin cytoskeleton at 20 min.

**Figure 2:**
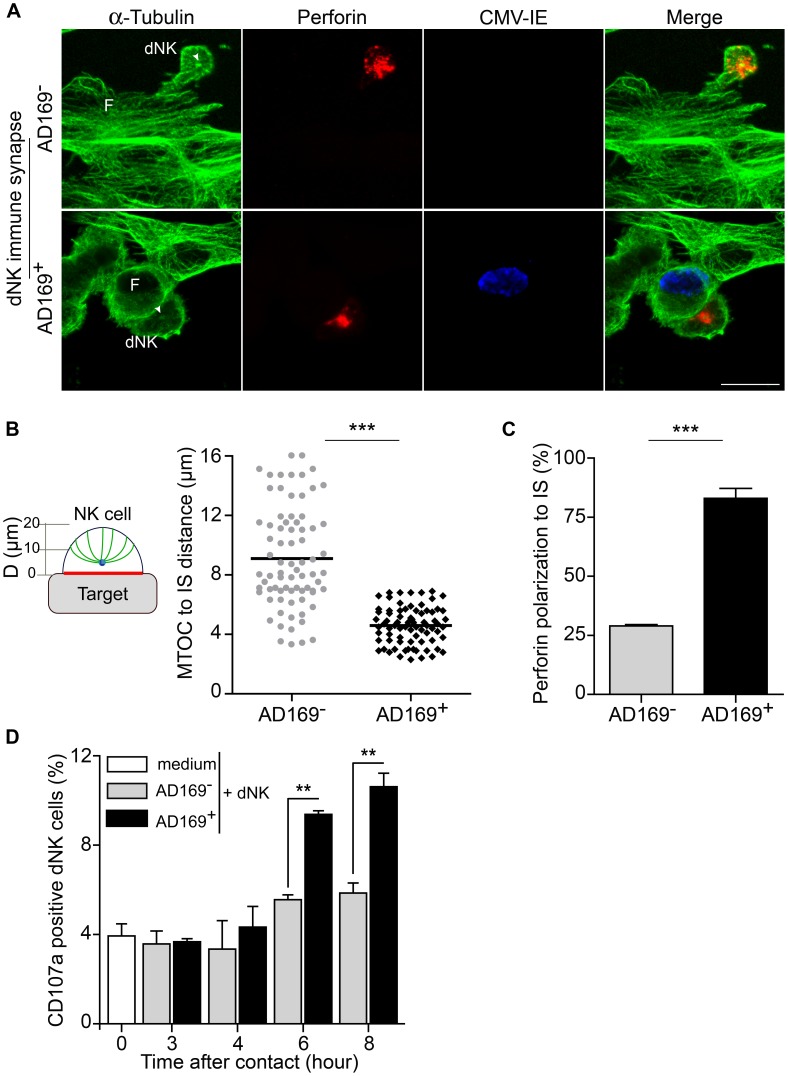
Polarization of the MTOC and lytic granules to the immune synapse formed with HCMV-infected fibroblasts. Uninfected (AD169^−^) or HCMV-infected (AD169^+^) decidual fibroblasts (F) plated on glass coverslips were incubated with autologous dNK cells (dNK) for 20 min at 37°C. (A) Representative images of maximum intensity projection. Microtubules (α-tubulin in green), lytic granules containing Perforin (red), HCMV-IE antigen (blue). Arrowhead points to the MTOC polarization (aster). Bar represent 20 µm. (B) Left cartoon shows schematic representation of the immunological synapse (IS), D (distance in µm). The center of IS was defined as the center of the contact zone between dNK and target cell (see cartoon, red line). Zero on the Y axis (µm) represents synaptic area; blue dot represents the microtubule organizing center (MTOC) and microtubules are in green. The MTOC polarization (Right graph) defined by the distance between the MTOC and the center of IS formed with uninfected (AD169^−^) and HCMV-infected (AD169^+^) fibroblasts. Distances were calculated for 50 conjugates from five independent experiments. Statistical analysis was performed using unpaired Student's *t*-test. ***, *p*<0.001. (C) Percentage of conjugates showing polarized perforin containing granules to the NKIS. Results from 5 independent conjugations were averaged, values represent means and S.D.s. At least 300 conjugates were analyzed. Statistical analysis was performed using unpaired Student's *t*-test. *** *p* = 0.0002. (D) Kinetic of CD107a cell surface expression was analyzed by flow cytometry on dNK cells that were in contact with uninfected or AD169-infected autologous fibroblasts. Values presented in the bar graphs are mean values calculated from three independent experiments done in triplicates at the ratio 1 to 1. Error bars are SEM. Statistical comparisons were performed using unpaired Student's *t*-test, ** *p*<0.01.

Being critical for the trafficking and delivery of lytic granules to the IS in NK cells [35], [36], we then analyzed the microtubule organizing center (MTOC) (Figure 2A) and the Golgi apparatus polarization (Figure S3A) in fixed conjugates after 20 min of interaction. dNK cells in contact with uninfected cells displayed a random localization of the MTOC (Figure 2A). In contrast, the majority of conjugates formed with HCMV-infected targets displayed a reoriented dNK cell MTOC towards the immune synapse (Figure 2A). We then finely defined the MTOC reorientation by measuring the distance between dNK cell MTOC and the center of the IS for each conjugate (defined as the center of the interaction zone dNK cell-target, see scheme Figure 2B). The distance between the MTOC and the center of IS showed a quite compact distribution in dNK cells that contacted AD169-infected fibroblasts with a mean distance of 4.6±1.25 µm (mean ± S.D.) (Figure 2B). In contrast, the distance from the MTOC to the center of the contact zone was very variable in dNK cell that formed conjugates with uninfected cells (Figure 2B) with a mean distance of 9.1±3.4 µm (mean ± S.D.).

We next analyzed the distribution of lytic granules containing perforin after 20 min of conjugation (Figure 2). Similar to the MTOC, perforin containing granules were localized in a random manner, but upon recognition of HCMV-infected cells, dNK cells polarized their perforin containing granules with the MTOC close to the contact zone (Figure 2A). Quantification of perforin polarization in a large number of immune synapses, demonstrated that while the majority of dNK cells that formed immune synapse with AD169-infected fibroblasts showed polarization of their lytic granules (83±4%), only 28% of dNK cells showed polarization towards uninfected targets (Figure 2C). Interestingly, when using a mixture of infected and non infected cells (one to one ratio), dNK cells polarize their MTOC and secretory machinery preferentially towards HCMV-infected fibroblasts (data not shown). Consistent with the MTOC and lytic granules, the Golgi apparatus was also distributed in clusters close to the MTOC only in dNK cells that formed immune synapses with AD169-infected fibroblasts (Figures S3A), but not in those that formed conjugates with uninfected targets (Figure S3A see right enlargement panels).

One of the critical step in the NK-IS formation includes the clustering of specific receptors that contribute to NK cell activation [34]. Despite the fact that CD9 would have been a better choice as it is mainly expressed by dNK cells but not pNK, decidual fibroblasts (data not shown) and other human fibroblasts express substantial amounts of this receptor [37], [38] we choose to analyze the localization of CD2 receptor for two main reasons. CD2 is expressed on the majority of dNK [9], [21] and it has been shown to rapidly cluster at the NK-IS [34], [39], [40]. Confocal analyses revealed that CD2 receptor microclusters were concentrated at the intercellular contact zone only in dNK cells that formed conjugates with infected fibroblasts (Figure S3C). We did not observe any changes in CD56 localization (data not shown). Thus, dNK cells engage mature immune synapse with HCMV-infected autologous fibroblasts that is characterized by polarization of the MTOC, the secretory machinery and clustering of CD2 activating receptor at the intercellular contact zone.

We next examined whether dNK cells were able to degranulate upon recognition of HMCV-infected fibroblasts by analyzing the cell surface expression of the Lysosomal-associated membrane protein 1 (LAMP1/CD107a) (Figure 2D). The kinetics of CD107a cell surface expression by dNK cells in contact with HCMV-infected autologous fibroblasts was carried out for 8 hours. Very little variations were observed within the first four hours of contact. After six hours of contact, a significant increase of CD107a expression was observed in dNK cells that are in contact with HCMV-infected autologous fibroblasts. The degranulation reached maximal level by 8 hours of contact (Figure 2D). The significant increase in CD107a cell surface expression indicates that IS formation is accompanied by efficient release of lytic granules and that dNK cells cytotoxicity is perforin-dependent but only after six hours of contact.

Collectively, these findings indicate that dNK display cytotoxic activity towards HCMV-infected autologous decidual fibroblasts but also emphasize the unique properties of dNK cells cytotoxicity. Even if dNK cells can form mature IS within normal range of time they do need extended time frame in order to release their lytic granules and perform efficient killing of HCMV-infected autologous fibroblasts.

### HCMV infection modulates dNK cell receptor repertoire

**Figure 3:**
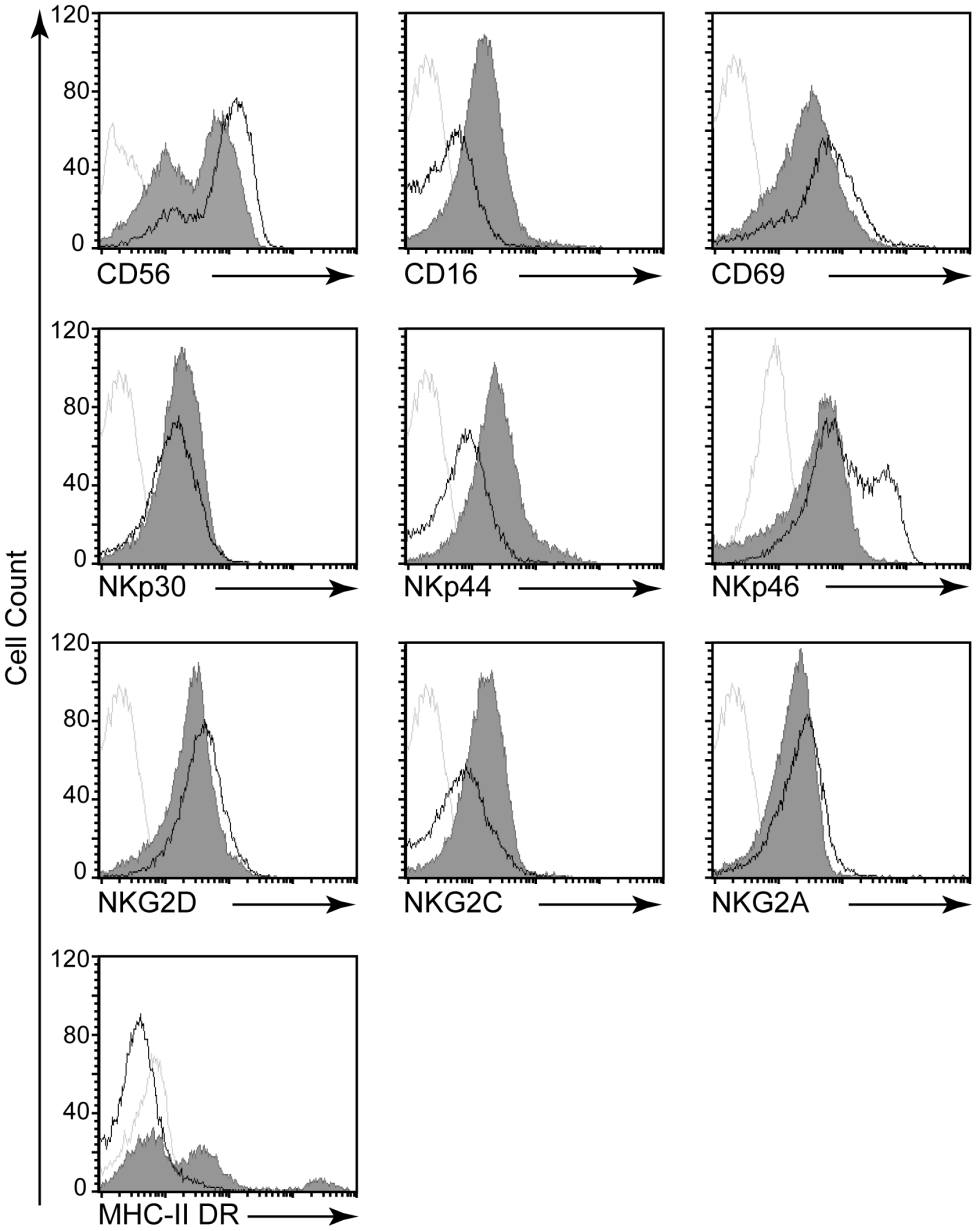
Exposure to infected cells modulates dNK cell receptor repertoire expression. dNK cells were co-cultured with autologous fibroblasts that were either uninfected or infected with HCMV-AD169 for 48 h. dNK cells were stained for surface expression of the indicated receptor using fluorochrome-conjugated antibodies and analyzed by flow cytometry. Representative FACS histograms gated on CD56^pos^ CD3^neg^ dNK cells are shown (n = 5). Specific receptors are indicated by the arrow. dNK cells in contact with uninfected fibroblasts are represented by black line, dNK cells in contact with HCMV-infected fibroblasts are represented by shaded gray. Dotted gray line represents isotype-matched control Ig. One representative histogram out of five independent experiments is shown.

The repertoire of NK activating and inhibitory receptors (NKRs) plays a critical role in cytotoxic activity of pNK cells and modulation of NKRs expression by these cells is often associated with their response to HCMV [30]. Thus, to provide further insights into the mechanisms involved in dNK cell cytolytic activity against HCMV, we analyzed whether these cells modulate their NKRs repertoire upon recognition of infected fibroblasts (Figure 3). Similar to freshly isolated dNK cells (data not shown), more than 76.3±5% (mean ± S.D.) of dNK cells co-cultured with uninfected autologous fibroblasts were CD56^bright^ (Figure 3). Exposure to HCMV-infected fibroblasts significantly decreased the percentage of CD56^bright^ dNK cells (48±6.3%), but significantly increased the percentage of CD56^dim^ cells (40±4%). The dampening down of CD56 expression was observed even after 18 hours of contact (Figure S4B) consistent with the acquisition of the cytotoxic profile. The changes in CD56 expression profile is always associated with the acquisition of CD16 expression (41% compared to 4.3%). There was a slight decrease in the mean fluorescence intensity of CD69 but the absolute number of CD69^pos^ dNK cells (85±5%) did not vary after contact with HCMV-infected fibroblasts. Although optimal changes were reached by 48 h, our data demonstrate that HCMV infection orchestrate dampening of CD56 and increase of CD16 expression observed as early as 18 h of contact (Figure S4B) which is consistent with acquisition of a cytotoxic profile.

To further characterize phenotypic changes in dNK cell receptor repertoire, we analyzed the expression of natural cytotoxicity receptors (NCRs) (NKp30, NKp44, and NKp46), NKG2D that recognize viral or stress induced ligands and NKG2A or C receptors that are expressed by a large fraction of dNK cells and recognize HLA-E molecules (Figure 3). The frequency of dNK cells expressing NKp44 activating receptor was significantly increased in dNK cells that were exposed to HCMV-infected fibroblasts as compared to those exposed to uninfected fibroblasts (90% versus 46%). Furthermore, co-culture with infected cells also induced major changes in the expression of NKp46 receptor. A significant shift in the fluorescence intensity towards an NKp46^low^ profile with a complete loss of the bimodal NKp46^hi^ and NKp46^low^ expression pattern was observed when dNK cells were exposed to HCMV-infected cells. More than 80% of dNK cells become NKG2C^+^ after their exposure to HCMV-infected fibroblasts, while only minor yet reproducible decreases in the percentage of NKG2A^+^ cells was observed. Exposure to HCMV-infected cells induced significant decrease in the percentage of cells expressing KIR2DL1, KIR2DL4 and ILT-2, while no changes were observed with those expressing KIR2DL2/3 (Figure S4A). Some of the changes in the expression of dNK cell repertoire were observed after 18 hours of contact (Figure S4B) while only discrete changes were observed for pNK cells (Figure S4C) further highlighting the originality of dNK cells and stretching their differences compared to pNK cells. Altogether, our data indicate that HCMV infection induces major changes in dNK cell receptor repertoire with increases in NKp44, NKG2C and decreases in NKp46, KIR2DL1, KIR2DL4 and ILT2 expression.

It has been suggested that *de novo* expression of MHC-II by NK cells and their acquisition of an APC-like phenotype could regulate the activation of numbering immune cells in particular T cells. Therefore, to further examine the modulation in dNK cells properties and phenotype upon exposure to HCMV-infected fibroblasts we examined the expression of HLA-DR in dNK cells co-cultured with infected and non-infected cells (Figure 3). A great fraction of dNK cells exposed to HCMV-infected fibroblasts, but not uninfected cells, acquired significant *de novo* expression of MHC-II DR at their cell surface (48%) displaying a bimodal distribution of fluorescence with a prevalence of positive cells expressing intermediate levels of these cell surface molecules. The acquisition of HLA-DR expression was effective even after 18 hours of contact (Figure S4B). Increases of HLA-DR expression were also observed in pNK cells that were in contact with HCMV-infected autologous fibroblasts (Figure S4C).

Taken together, these data show that exposure to HCMV-infected fibroblasts not only modulates dNK cell receptor repertoire but also increases the expression of key elements of adaptive response (HLA-DR).

### NKG2D and CD94/NKG2 activating receptors modulate dNK cell responsiveness to HCMV-infected fibroblasts

**Figure 4:**
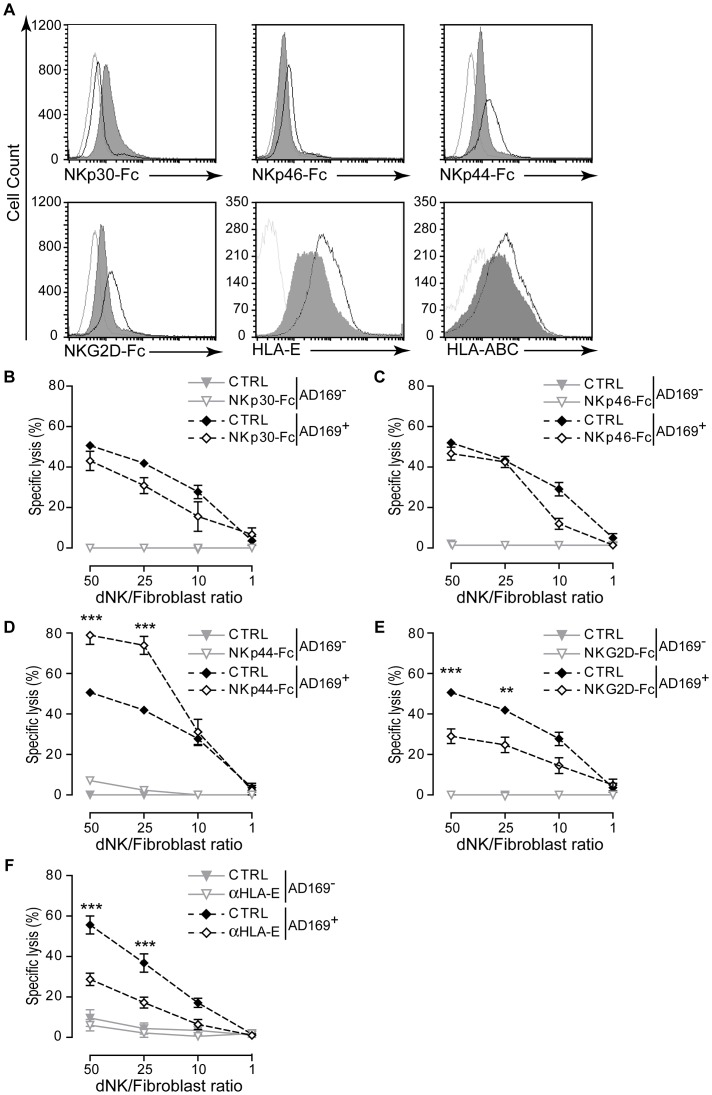
Functional analysis dNK cells specific receptors. (A) HCMV-infection modulates the expression of NKR ligands on decidual fibroblasts. The binding of human NKp30-Fc, NKp46-Fc, NKp44-Fc, and NKG2D-Fc chimera was used to evaluate the cell surface expression of specific receptor ligands. The expression of HLA-E and HLA-A,-B,-C molecules was evaluated using specific mAb. Uninfected fibroblasts are represented by black line, HCMV-infected fibroblasts (shaded gray). Dotted gray line represents represent negative control or isotype-matched control Ig. One representative FACS histogram out of five independent experiments is shown. (B–E) Decidual fibroblasts uninfected (AD169^−^) or infected (AD169^+^) were incubated with soluble receptor-Fc fusion protein at the concentration of 1 µg/ml and dNK cell cytotoxicity was analyzed by chromium release assay after 18 h of co-culture. Control lysis was performed in the presence of CD99-Fc chimera (CTRL). Lysis analyzed in the presence of (B) NKp30-Fc, (C) NKp46-Fc, (D) NKp44-Fc, (E) NKG2D-Fc. (F) Analysis of NKG2A and NKG2C function was performed in the presence of blocking antibody against HLA-E molecules (α-HLA-E). Control lysis performed in the presence of isotype match control Ig (CTRL). Data sets represent mean lysis ± S.D. from five independent experiments done in replicate. Statistical comparisons were performed using two-way ANOVA test. ***, *p*<0.001; **, *p*<0.01.

Cytotoxic function of NK cells could involve several NKRs. To provide insights to their possible involvement in dNK cell cytotoxicity against HCMV-infected fibroblasts, we took advantage of Fc-chimeras to analyze NKR ligands expression in uninfected, AD169-infected (Figure 4A), or VHLE-infected (Figure S5A) decidual fibroblasts. Uninfected fibroblasts expressed low levels of NKp30L. Similar to human fetal foreskin fibroblasts (HFFF) [41], HCMV infection led to an increase in NKp30L expression by decidual fibroblasts (Figure 4A, S5A). Decidual fibroblasts expressed low levels of NKp46L that was further decreased after HCMV infection. By contrast to NKp30L, ligands for NKp44, and NKG2D were highly expressed in uninfected decidual fibroblasts (Figure 4A, S5A). Both HCMV strain induced significant decreases in the expression of NKp44L and NKG2DL (Figure 4A, S5A).

We then investigated whether HCMV infection affected the expression level of HLA-E cell surface molecules. As shown in figure 4A (and S5A), decidual fibroblasts expressed both the nonclassical HLA-E and the classical HLA-A,-B,-C molecules at their surface. While infection with HCMV resulted in a significant decrease in HLA-E expression, only small effect was observed for the expression of classical HLA-A,-B,-C. This striking observation of HLA-E downregulation by HCMV prompted us to perform further analyses comparing the impact of HCMV infection in additional decidual fibroblasts and in other cells (Figure S5B). Consistently, we observed downregulation of cell surface expression of molecules HLA-E in HCMV-infected decidual fibroblasts (Figure 4, S5A, S5B). Consistent with previous studies using HFFF cells [42], [43] and in contrast to decidual fibroblasts, HCMV resulted in upregulation of cell surface HLA-E in MRC-5 fibroblasts and in HEK293T cells (Figure S5B and data not shown). We also observed a small decrease in the level of HLA-A,-B,-C in these cell lines (Figure S5B and data not shown). Western blot analyses of total amount of HLA-E molecules demonstrated that HCMV-infection did not affect total amount of HLA-E proteins in decidual fibroblasts while increased levels were observed in MRC-5 cells expression (Figure S5C). The CD94/NKG2X (-A, -C or -E) family members recognize HLA-E molecule but these receptors can transmit opposing signals [23], [44], [45]. The differences between the two systems imply that HCMV infection of decidual fibroblasts might trigger their recognition and promote their killing through engagement of CD94/NKG2C/E activating receptors. This is in line with observed up-regulation of NKG2C on dNK upon their recognition of infected decidual fibroblast (Figure 3) and the high levels of NKG2E on dNK cells [12].

Using Fc-chimeric proteins to block specific receptor/ligand interactions, we found that neither blockade of NKp30 (Figure 4B) nor of NKp46 (Figure 4C), both modulated upon HCMV infection, interaction with their putative ligand(s) had an effect on dNK cell killing of autologous HCMV-infected fibroblasts. Blocking the interaction of NKp44 activating receptor with its ligand resulted in 50% increased killing of infected autologous fibroblasts (Figure 4D). In contrast, interference with NKG2D receptor ligation induced a significant decrease in dNK cell cytotoxicity; the mean lysis of HCMV-infected fibroblasts was 50% whereas only 20% of infected cells were lysed in the presence of NKG2D-Fc chimeric protein (Figure 4E). The decrease in cytotoxicity after blockade of NKG2D ligation to its cognate ligands expressed on HCMV-infected fibroblasts underscored a role for NKG2D receptor in dNK cell cytotoxicity. Since neither NKp30-Fc nor NKp46-Fc had an effect on dNK cells lysis, we tested the ability of these chimeras to block pNK cell cytotoxicity. The binding of either chimera significantly decreased the killing of K562 cell line by pNK cells (Figure S6A) indicating that both chimeras are functionally active.

Since HLA-E is a ligand for both inhibitory CD94/NKG2A and activating CD94/NKG2C/E receptors, we explored its involvement in dNK cell cytotoxic response against HCMV-infected fibroblasts. To this end, we performed lysis assay in the presence of an anti-HLA-E blocking monoclonal antibody. Blockade of HLA-E ligation with its cognate receptor resulted in a two-fold decrease of the sensitivity to dNK cell lysis (36% compared to 75% for IgG1 isotype control) (Figure 4F). This inhibitory effect suggests that in our system model, HLA-E on infected-fibroblasts binds to the CD94/NKG2C or -E activating receptors rather than to CD94/NKG2A inhibitory receptor and such binding could mediate the cytotoxic effect of dNK.

Examination of pNK cell cytotoxicity shows that even though some minor changes were constantly observed with NKp30, NKp46 and NKp44 receptors (Figure S6B–D), only the NKG2D receptor played a major role in the killing of HCMV-infected autologous decidual fibroblasts (Figure S6E) as its blockade resulted in significant decrease in pNK cell cytotoxicity against autologous fibroblasts. The blockade of HLA-E did not impair pNK cell cytotoxicity (Figure S6F).

Taken together, our data uncover a crucial role of NKG2D and CD94/NKG2C or -E activating receptors in dNK cell cytotoxic response against HCMV-infected fibroblasts, while neither NKp30 nor NKp46 are implicated in dNK cell response. By contrast to its activating role in peripheral blood NK [24], NKp44 have an inhibitory effect in the control of dNK cell cytotoxic function.

### Exposure to infected fibroblasts modulates dNK cells cytokine and chemokine secretion

**Figure 5:**
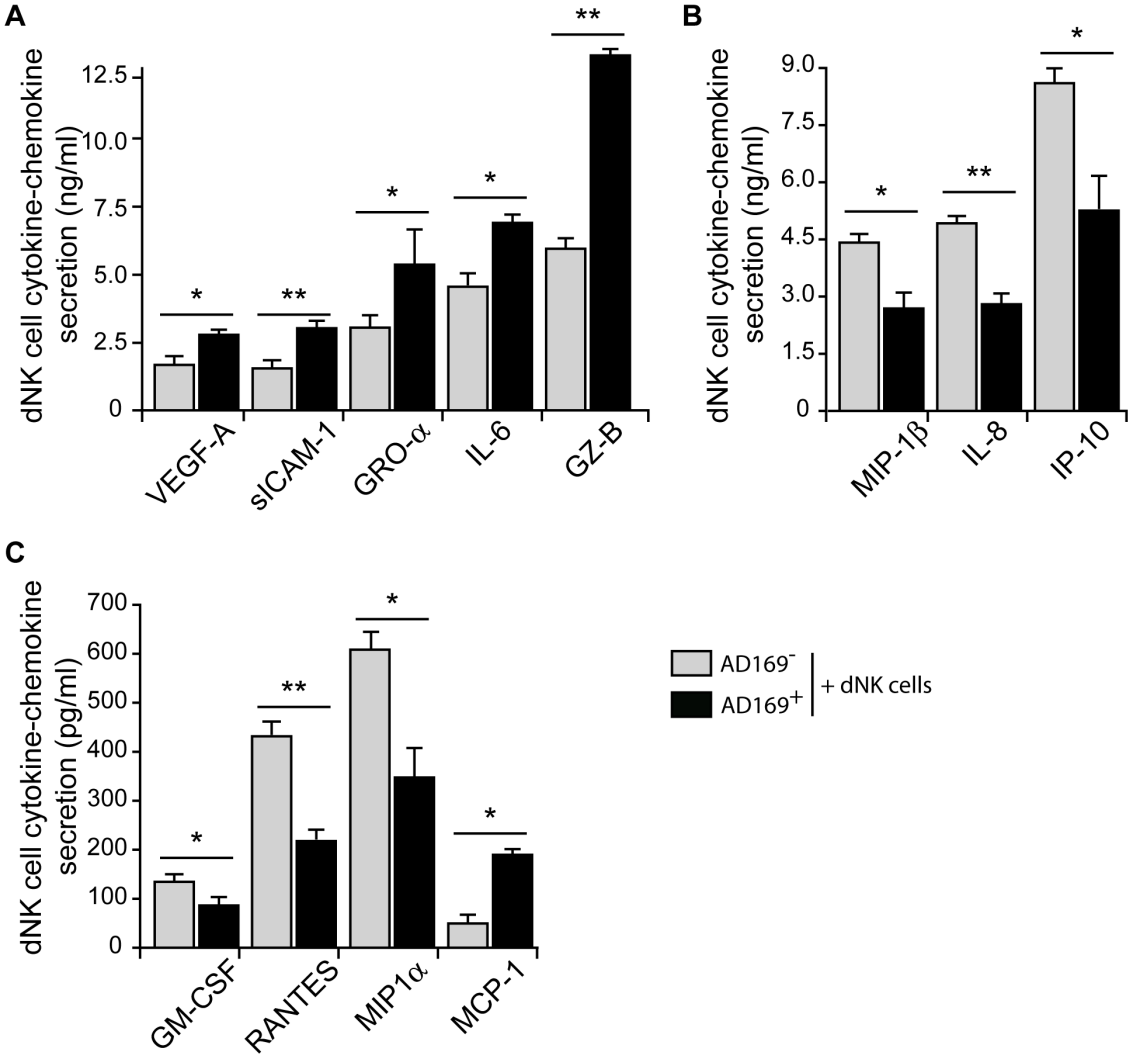
HCMV infection modulates dNK cells cytokine/chemokine production. dNK cells were stimulated with uninfected (AD169^−^, gray) or AD169-infected (AD169^+^, black) autologous decidual fibroblasts for 24 h. Cytokines were quantified in the supernatants using a 42-multi-plexed cytokine assay. Representative histograms from selected cytokines-chemokines that showed significant differences are presented. (A & B) Soluble factors that are produced at high levels by dNK cells. (C) Soluble factors that are produced at low levels by dNK cells. Concentrations are given as differences between secretions of dNK cell in presence of uninfected or infected fibroblasts and the corresponding uninfected or infected fibroblasts. Normalized data points are given as mean ± S.D. calculated as from four independent experiments. Statistical comparisons were performed using Mann & Whitney test. **, *p*<0.01; *, *p*<0.05.

In normal pregnancy, dNK cells are known to secrete great amount of soluble factors that play a key role in trophoblast attraction and vasculature remodeling. Since some of dNK cell soluble factors have also been found in HCMV secretome [46], we first analyzed the secretion profile of uninfected and HCMV-infected decidual fibroblasts, using a 42-multiplexed cytokine/chemokine/growth factor Luminex assay (Figure S7). Decidual fibroblasts produced GRO-α/CXCL-1, sICAM-1, IL-6, IL-8, IP10, MCP-1, MIP1β, MIP1β, and VEGF-A. After HCMV-infection, mild variations were observed for IL-8, MIP-1β and VEGF-A without reaching statistical significance and only IL-6 secretion was significantly increased in AD169-infected decidual fibroblasts (1.7 fold increase) (Figure S7). To examine whether HCMV infection modulates dNK cell secretion profile, we analyzed specific dNK cell secretion in co-cultures either with uninfected or HCMV-infected autologous decidual fibroblasts (Figure 5). Although large variations were observed amongst the donors, only a limited number of secreted cytokines, chemokines and growth factors varied after 24 h of co-culture with HCMV-infected autologous targets (Figure 5). Similar to freshly isolated dNK cells ([9] and data not shown), dNK cells that were in contact with autologous uninfected decidual fibroblasts produced VEGF-A, sICAM-1, GRO-α/CXCL-1, IL-6, Granzyme B (GZ-B) (Figure 5A), MIP-1β/CCL4, IL-8/CXCL8 and IP-10/CXCL10 (Figure 5B). They also produced substantial amounts of GM-CSF, RANTES/CCL5, MIP-1α/CCL3 and low amounts of MCP-1/CCL2 (Figure 5C). Stimulation of dNK cells with HCMV-infected fibroblasts led to significant increased secretion of VEGF-A (1.6-fold), sICAM-1 (1.7-fold), GRO-α/CXCL-1 (2-fold), IL-6 (1.5-fold), GZ-B (2.1-fold) (Figure 5A) and MCP-1/CCL-2 (3.5-fold) (Figure 5C). On the other hand, the production of MIP-1β, IL-8, IP10 (Figure 5B), GM-CSF, RANTES, MIP-1α (Figure 5C) was significantly decreased after stimulation with HCMV-infected cells. Finally, all other cytokines and chemokines tested were either below cut-off levels (IFN-γ, IFN-ω, TGF-α, TNF-α/β, IL-1β, IL-2, IL2RA, IL-4, IL-5, IL-10, IL-12, IL-15, IL-17A/F, EGF, E-Selectin and Leptin) or did not vary after exposure to HCMV-infected fibroblasts (basic FGF, IFN-α2, IFN-β, IL-1α, IL-1RA, IL-22, SDF-1, sFas, sFasL, TRAIL, Eotaxin-3/CCL26, Fractalkine/CX3CL1) (data not shown). Overall, these data demonstrate that HCMV-infection modulates the secretory profile of dNK cells, with increased production of cytotoxic factors that may constitute virus-specific immune response.

### dNK cells infiltrate HCMV-infected trophoblast

**Figure 6:**
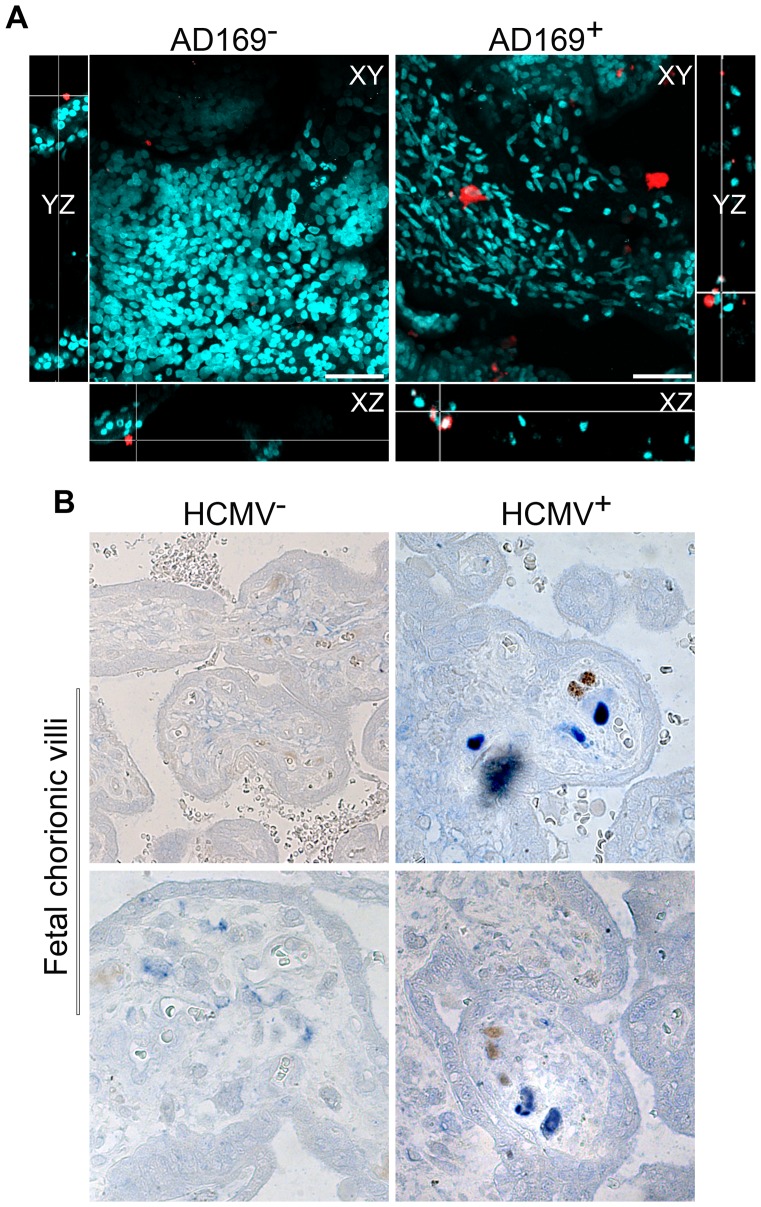
dNK cells infiltrate infected placental tissues. (A) Two-color 3D-images of chorionic *villi* explants organ cultures established from first trimester trophoblast either uninfected (AD169^−^) or HCMV-infected (AD169^+^). Nuclei were stained with dapi (cyan). Infiltrating dNK cells (red). Lower and side panels show orthogonal XZ and YZ slices, respectively. Images are from two-photon Z-stack (total of 200 µm). Scale bars = 100 µm. (B) Two-color IHC of 6-µm-thick sections from paraffin-embedded whole placental biopsies of HCMV^+^ pregnancy termination. HCMV^+^ and HCMV^−^ tissue sections are presented. Representative immunostaining of HCMV-IE (blue, alkaline phosphatase staining) and CD56^+^ NK cells (brown, peroxidase staining) (n = 2).

The maternal *decidua* is the main fetal-maternal interface where maternal dNK cells are in close contact with invasive fetal trophoblast. HCMV virions are believed to disseminate from decidual cells to the invasive trophoblasts and in floating and anchoring villous trophoblasts [5]. To support the relevance of our results, we developed an organ culture model of trophoblastic *villi* explants to assess the ability of dNK cells to infiltrate infected tissues. Villous explants were isolated, infected (48 h) or not and cultured for 2 h with autologous dNK cells that were labeled with CellTraker Red. As shown in Figure 6A and supplementary movies very few dNK cells were able to establish contact with autologous uninfected trophoblast. However, large number of dNK cells was able to infiltrate and establish close cellular contacts within HCMV-infected organ explants. We were able to analyze organ culture over 250 µm deep section and demonstrate that dNK cells were able to formed synapse like structures with infected cells throughout the section (see 3D-reconstitution movie). These data demonstrate that dNK cells are able to sense and migrate within the infected tissues.

We then investigated the ability of dNK cells to interact with infected tissues *in vivo*; we analyze biopsies of placental samples from 24–26 weeks HCMV^+^ termination of pregnancy (Figure 6B). Thin sections of placental samples were analyzed by IHC for the presence of NK cells using anti-CD56 marker and anti-CMV-IE antibodies. Analysis of infected placenta showed that CD56^pos^ cells were present at the vicinity of infected HCMV positive cells (Figure 6B) while no CD56^pos^ cells were present in the HCMV negative tissue. Together these results clearly demonstrate that dNK (CD56^pos^) cells are able to infiltrate HCMV-infected tissue both *in vitro* in organ culture model and *in situ* within HCMV^+^ placentas, providing thus solid evidence for the implication of dNK cells in controlling HCMV infection and spreading.

## Discussion

Despite their importance in maintaining healthy pregnancy, the control of maternal HCMV infection and spreading by dNK cells is not yet fully understood. Our study is the first to assign a critical role to dNK cells in controlling maternal HCMV infection and in limiting its spreading to fetal tissues through their capacity to acquire potent cytotoxic activity when in contact with infected decidual cells. During normal pregnancy, the majority of dNK cells are CD56^bright^CD16^neg^. They secrete a large panel of cytokines and chemokines that are necessary for placental development. We demonstrate that dNK cells undergo phenotypic and cellular changes that allow them to recognize and kill autologous HCMV-infected cells in a FasL- and TRAIL-independent manner.

Immunological synapse formation is a crucial step for the delivery of lethal hits by effector cells. Rapid re-localization of the MTOC is needed for the trafficking and the polarization of lytic granules to the IS [34], [47]–[50]. We show that although dNK cells recognize and engage IS with HCMV-infected cells very rapidly, they require longer exposure time in order to degranulate and exert the cytotoxic effector function. The delay to unleash dNK cell cytotoxic effector function might correspond to the time necessary for dNK cells to mature and acquire necessary functional changes to exert cytotoxicity. However, we cannot exclude that HCMV-infected fibroblasts provide weak signal to induce fast degranulation or that decidual fibroblasts have an inherent resistance to cytotoxic granule mediated cell death.

Mechanisms that prevent dNK cell cytotoxicity are not completely understood. Even though dNK and pNK cells exhibit similar expression levels of cytotoxicity encoding genes [12], under healthy conditions dNK cells are tolerant to semi-allogeneic fetal trophoblasts. Although mechanisms that control cytotoxicity are not well established, they may include strong interactions of inhibitory receptors with their cognate ligands expressed by fetal trophoblast, production of VEGF-C by dNK cells and/or expression of anti-apoptotic proteins (XIAP) by target cells [51], [52]. The lack of dNK cell cytotoxicity can be reversed, at least *in vitro*, after exposure to cytokines such as IL-5 and IL-18 or upon engagement of specific activating receptors [9], [21]. Here we show that HCMV infection provides the necessary activating signals to trigger dNK cell cytotoxicity. The fact that dNK cells killed heterologous targets from a different donor further emphasizes the intrinsic ability of these cells to kill when they are exposed to the right activating signals. Our observation that dNK cells did not kill semi-allogeneic trophoblasts but killed HCMV-infected autologous fibroblasts highlights their plasticity and their specific ability to respond to HCMV infection.

In contrast to pNK cells, very little is known about dNK cell cytotoxicity as these cells are mainly cytokine and chemokine producers [9], [10], [13], [21]. We demonstrated that under HCMV-infectious conditions, a significant fraction of dNK cells that are CD56^bright^ and CD16^neg^ rapidly dampened down their CD56 expression level and acquired CD16 expression. These changes are most probably due to the acquisition of cytotoxic function. Several NKRs have been involved in pNK cell cytotoxicity [53]. For instance, efficient control of HCMV infection involves NKG2D receptor and can be associated with the emergence of NKG2C^+^ subset that contribute to long term protective immune response [30]. Exposure of dNK cells to HCMV-infected fibroblasts resulted in an increased NKG2C^+^ expression without major changes in NKG2A expression. The role of other receptors in NK cell response to HCMV is not completely understood. HCMV is able to decrease a plethora of key receptor-ligand interactions that are involved in NK-cell response. By contrast to changes in pNK cell repertoire [22], opposite effects were observed for NKp44 and NKp46 receptors while no changes were observed for NKp30 receptor. These observations further highlight differences between dNK and pNK cells *modi operandi* during HCMV infection.

Since the nature of HCMV-induced cellular ligands is not known, we took advantage of NKR-Fc chimeric receptors to analyze the expression of NKR ligands on decidual fibroblasts. Although some variations were observed amongst different *decidua basalis*, we found that decidual fibroblasts constitutively express ligands for NKp44 and NKG2D while they barely express ligands for NKp30 or NKp46. HCMV infection induced NKp30L and resulted in significant decreases of NKp44L and NKG2DL but did not affect the expression of NKp46L. These findings suggest that HCMV infection interferes with the expression level of activating receptor ligands even if some of them are of cellular rather than virally induced.

Using chimeric proteins, we demonstrated that NKp44 receptor plays an inhibitory function in dNK cell cytotoxicity. dNK cells might express an inhibitory isoform of NKp44 receptor as a result of *NCR2* alternative splicing as it has been recently demonstrated for *NCR3* (NKp30) [54]. Alternatively, NKp44L expressed on decidual fibroblasts might participate to uncoupling of activating adaptor molecules thus promoting an inhibitory profile. However, the expression of an inhibitory isoform is the most likely explanation since dNK cells constitutively express the NKp44 receptor. It has been clearly demonstrated that HCMV maintains an inhibitory status either by preventing the cell-surface expression of NKG2D activating ligands [55], [56] or by UL40-mediated up-regulation of HLA-E or MHC-I like surrogates molecules expression. Although, there are some discrepancies between our two observations, namely decreases of NKG2DL and acquired cytotoxicity through NKG2D receptor, it is possible that decreases in NKG2DL are selective resulting in the expression of high affinity ligands. Alternatively, co-engagement of other activating receptors is sufficient even if there is less NKG2D ligands.

Further studies are needed to identify NKG2DL that are expressed on decidual cells. Discovery of such ligand and the characterization of specific receptor-ligand interactions that mediates dNK cellular cytotoxicity will help uncover potential therapeutic target that, when activated *in vivo*, can limit viral spreading and/or prevent congenital disease.

Previous investigations demonstrated that both classical and non-classical MHC-I molecules have been targeted by HCMV evasion strategies. By contrast to human fetal foreskin fibroblasts and fibroblastic cell lines [42], [43], HCMV-infection resulted in decreased cell surface HLA-E molecules without affecting the total amount of proteins in decidual fibroblasts. The difference between the two cellular systems might reside in the fact that decidual fibroblasts express substantial amounts of HLA-E at the steady state. In decidual fibroblasts, HCMV might interfere with the stability of cell surface HLA-E molecules by impairing rapid protein export or by increasing intra-cellular retention. The inhibitory profile observed upon blockade of HLA-E in HCMV-infected fibroblasts further support the involvement of CD94/NKG2C or possibly CD94/NKG2E activating receptors, both greatly expressed by dNK cells [22]–[31]. In this context, HCMV peptides might play a critical role in promoting the recognition of HLA-E by activating members of CD94/NKG2C and CD94/NKG2E receptors thus increasing susceptibility of decidual fibroblasts to dNK cell cytotoxicity at early times of infection as it has been shown previously for pNK cells [57]. It will be very interesting to investigate whether late HCMV infection is responsible for similar changes and whether specific HCMV peptides play roles in the sequential changes in dNK cell function.

In parallel to these changes in NK cell receptor, dNK cells acquire *de novo* expression of MHC-II DR molecules. This potential acquisition of an APC-like phenotype during the course of HCMV immune response might play a crucial role in initiating a cross-talk with neighboring immune cells, including CD4^+^ T cells. Indeed, within the fetal-maternal interface, dNK cells are in close proximity with decidual CD4^+^ T cells. Expression of MHC-II DR antigens might be necessary for dNK cell activation and for shaping up the adaptive immunity [58], [59]. However, further investigations are needed to demonstrate whether the expression of MHC-II molecules is associated with the acquisition of APC capabilities and HCMV antigen presentation.

It is very intriguing that only few cytokines and chemokines varied in the presence of HCMV infected fibroblasts. HCMV infection induces IL-6 secretion most probably through the expression of the viral-encoded chemokine receptor US28 and the activation of the IL6/STAT3 signaling pathway [60]. Interestingly, IL-6 was further increased when dNK cells were in contact with HCMV-infected fibroblasts, most probably through a paracrine effect on dNK cells. sICAM-1 was also increased under HCMV conditions. Previous reports suggested that IL-6 down-regulates the production of several soluble factors [61], while sICAM-1 increases have been correlated to HCMV reactivation [62]. Both IL-8 and IP-10 are necessary for trophoblast migration as these cells express a panel of receptors allowing them to respond to these chemokines [10]. By lowering the level of IL-8 and IP-10, dNK cells might reduce trophoblast invasion and prevent viral spreading from decidual stroma to fetal tissue or be partially responsible for fetal damages. Remarkably and in sharp contrast with pNK cell response to viral infection [63], [64], there were no changes in secretion levels of cytokines such IL-12, IL15, type I IFN, TNFα or IFN-γ that are all known to regulate NK cell function. Moreover, it is possible that changes in dNK cell secretome create the necessary inflammatory environment that will favor the recruitment and the initiation of anti-HCMV adaptive immune response.

We demonstrate that during HCMV infection, there is a bias of the inflammatory environment in the *decidua basalis*. dNK cells seem to lose their “decidual status” and become killers in order to limit viral infection. Exposure to HCMV infection can imprint dNK cell receptor repertoire towards killer activity. We demonstrate that NKG2D, NKG2C/E activating receptors play a crucial role in dNK cell cytotoxic response against HCMV-infected fibroblasts. The fact that dNK cells are able to infiltrate HCMV-infected tissue *in vitro* and engage immunological synapse-like structures within the infected placentas *in situ* strongly suggest that dNK cells are key players in controlling HCMV infection and spreading during pregnancy. To our knowledge, we provide for the first time evidence for the involvement of dNK cells in clearing HCMV infection. In fact, we clearly show that dNK cells that are present only in the *decidua basalis* during healthy pregnancy are in contact with HCMV-infected fetal tissue *in vivo*. It is possible that upon activation there is an increased dynamic of dNK cells allowing them to rich fetal site, which is normally devoid of maternal immune cells, and kill HCMV-infected cells. Recent reports have clearly linked the ability of NK cells in controlling HCV replication and liver fibrosis to specific soluble factor secretion and/or specific activating receptor expression [65], [66]. Future studies, with large cohort of placentas from medical termination of pregnancy due to congenital HCMV infection, will be necessary to clarify the dynamic of dNK cell activation *in vivo* as well as the pivotal role of soluble factor secretion in mounting proper anti-HCMV responses and limiting virus spreading.

In conclusion, our data shed new light on the plasticity of dNK cells and provide evidence for a correlation between phenotypic changes and functional anti-viral response. We have demonstrated the ability of dNK cells to exert anti-viral effector functions *in vitro* and to infiltrate HCMV infected tissues both *ex-vivo* and form immune synapse like-structures *in vivo*. Careful investigations of dNK cell status *in vivo* in larger cohorts of HCMV^+^ termination of pregnancy will be required to see whether this predicts clinical outcome. Understanding mechanisms that regulate switch in dNK cell immune tolerance will help us discover key factors/pathways that are involved in the immunopathology of HCMV infection during pregnancy and design strategies to limit congenital infection.

## Materials and Methods

This study was approved by the Research Ethical Comity Haute-Garonne. All patients signed the informed consent before samples were taken, Agence de la Biomédecine, PFS08-022.

### Cell purification, cell lines

dNK cells were purified from first-trimester *decidua basalis* (8–12 wk of pregnancy) obtained after elective pregnancy terminations as previously described [9]. Briefly, *decidua* samples were minced, collagenase IV treated. Cell suspension is then allowed to adhere in tissue culture plates. dNK cells were purified from the non adherent cell fraction using MACS negative selection kits according to the manufacturer procedure (Miltenyi Biotech). dNK cells were kept at 4°C in conditioned media containing 20% heat-inactivated fetal calf serum (FCS). Autologous fibroblasts were purified from the adherent mononuclear cell fraction by successive round of mild trypsin treatment.

The purity of both dNK cells and decidual fibroblasts was assessed using a cocktail of antibodies. dNK cells were CD3^neg^ and CD56^pos^. Decidual fibroblasts purity was confirmed by immunostaining with an anti-cytokeratin and anti-vimentin antibodies, fibroblasts are cytokeratin 7^neg^ (NM_001047870) and Vimentin^pos^ (NM_003380).

### Virus production and cellular infection

Decidual fibroblasts were maintained in RPMI-1640 medium (GIBCO) supplemented with 10% (v/v) FCS and penicillin-streptomycin 100 U/ml each, under a 5% CO_2_ atmosphere at 37°C.

Two HCMV strains were used, AD169 laboratory strain (ATCC strain, a gift from S. Michelson, Paris, France), and VHLE clinical isolate (a gift from C. Sinzger, Tubingen, Germany). Viral stocks were prepared from cell-released virions, using MRC-5 cells as previously described [67]. High titer virus stocks were stored in single use aliquots for up to six months at −80°C.

Adherent cell monolayers of decidual fibroblasts or MRC-5 cell line were infected with HCMV particles (MOI 3–5) for 48–72 hours. Trophoblastic villous explants were infected under the same conditions.

### Flow cytometry

Fibroblasts were cultured with dNK cells at 1∶1 ratio. After 48 h of co-culture, conjugates were disrupted mechanically by repeated pipeting and dNK cells were collected and washed twice in PBS. dNK cell suspension were then liquoted in 100 µl containing 1×10^5^ cells and labeled with fluorophore-conjugated antibodies. The following mAbs were used: CD56-APC, CD3-PE-Cy7, CD16-PE, CD69-PE, CD2-PE, NKG2D-PE, NKG2A-PE, HLA-DR-FITC, KIR2DL1-PE and 2B4-PE (BD Pharmingen, France); NKp30-PE, NKp44-PE, NKp46-PE, KIR2DL2/3-PE (Beckman Coulter, France); NKG2C-PE, KIR2DL4 clone 181703 (R&D Systems, France); ILT2-PE (Biolegend); CD107a-PE, anti-human HLA-I (HLA-A,-B,-C BC)-PE (BD Pharmingen) and matched isotype controls. Histograms shown were obtained by applying a gate on CD56^pos^ CD3^neg^ dNK cells.

Fibroblasts were detached using 0.05% trypsin-EDTA, washed twice in buffer containing 1% FCS. Cells (5×10^5^ to 10^6^) were resuspended in 100 µl of FCS containing buffer and incubated either with primary specific Ab or isotype matched control followed by mouse anti-human IgG1 FITC coupled Ab. The expression of NCR-ligands on fibroblasts was analyzed by binding of NCR-Fc chimera followed immunostaining with FITC-coupled mouse anti-human IgG1 secondary Ab (Southern Biotec). The following chimeras were used: NKp30-Fc, NKp44-Fc, NKp46-Fc and NKG2D-Fc, CD99-Fc (R&D Systems, France). Non specific binding was blocked by preincubating the cells for 30 min in 2% FCS and 1% BSA containing buffer. Data were analyzed using CellQuest (Becton Dickinson).

### Immunofluorescence, Conjugation and Confocal microscopy

For conjugation, fibroblasts were seeded onto 24-well plates containing glass coverslips. After 16 h adhesion, dNK cells were added at a 1 to 2 ratio and incubated at 37°C. Cells were washed briefly with PBS and fixed with 4% paraformaldehyde for 20 min and washed in PBS. Intracellular staining was in the presence of 0.5% Saponin. Cells were incubated in PBS containing 1% heat-inactivated calf serum for 30 min and stained with primary antibodies followed by incubation with Alexa fluor conjugated secondary antibody (Invitrogen) as previously described [68]. Filamentous actin cytoskeleton was visualized with Alexa fluor conjugated phalloidin. After extensive washing, coverslips were mounted with vectashield mounting medium (DAKO). Fluorescence was analyzed using Zeiss LSM710 confocal microscope using a 63x oil objective (Carl Zeiss AG, Jena, Germany).

Cell morphology was analyzed by examining the phalloidin-stained conjugates as an indicator of F-actin distribution. Images correspond to maximum intensity projection along the z-axis (Zen software).

The distance between the MTOC and the center of the IS was measured from single plane of unprocessed images using the single line function of the Imaris (Biplane Scientific Software).

The following antibodies were used to analyze microtubules and MTOC, perforin, CMV infection: anti-human alpha tubulin polyclonal Ab (Sigma-Aldrich, UK), anti-human golgin-97 (Invitrogen), anti-human perforin and anti-human CD2 (BD Pharmingen), anti-HCMV-IE (Argene), anti-human vimentin and anti-cytokeratin 7 (Dako). The F-actin cytoskeleton was analyzed using phalloidin coupled to either to Alexa fluor 488 or Alexa fluor 747 (Invitrogen).

### Degranulation assay, CD107a expression

For degranulation assay, fibroblasts were harvested and incubated at 37°C with dNK cells at a 1 to 1 ratio for different time points. Reactions were stopped on ice, cells were stained with fluorochrome-conjugated anti-human CD107a (BD Pharmingen) or isotype matched control Ab in staining buffer containing 1% FCS. Fluorescence was analyzed by Flow Cytometry.

### Cytotoxicity assays

Target cells (1×10^6^cells) were labeled with 100 µCi ^51^Chromium (Sodium chromate, 1 mCi/ml, Perkin Elmer, Courtaboeuf, France). After 1 h incubation at 37°C, cells were washed 3 times in HEPES-buffered RPMI. dNK effector cells were added to ^51^Cr labeled target cells (5×10^3^) in replicate at various effector to target ratios in a total volume of 200 µl RPMI containing 5% FCS per well of 96-well round-bottomed microtiter plates. Microtiter plates were centrifuged at 1200 rpm for 5 min and incubated at 37°C. After 4 h or 18 h of culture, 50 µl cell free supernatants were transferred to Lumaplate (Perkin-Elmer) and the radioactivity was measured on a TopCount (Perkin-Elmer). The specific cytotoxicity was calculated. Spontaneous release was determined from wells containing target cells alone. Maximum release was determined from wells containing target cells lysed in 1% Triton X-100. The data were expressed as follows:% specific cytotoxicity = 100×[Sample mean (cpm) - Spontaneous mean (cpm)/(Maximum mean (cpm) − Spontaneous mean (cpm)]. To block cell lysis due to the engagement of specific activating receptor engagement or specific pathway, ^51^Cr-labeled target cells were incubated for 20 min on ice with various soluble receptor-Fc IgG1 chimeric protein (0.2 µg/ml), anti-HLA-E mAb (clone MEM-E/08, Exbio) or an isotype match control (mouse IgG1) at the final concentration of 1.0 µg/ml then included as targets in cytotoxicity assay with dNK effector cells. dNK cells were incubated with an anti-TRAIL and -FasL antibodies (10 µg/ml) (R&D Systems, France) prior to cytotoxicity assay. Recombinant TRAIL and FasL proteins (gifts from A. Quillet-Mary, Toulouse, France) were used at the final concentration of 10 µg/ml.

### Mutiplex cytokine and chemokine arrays

dNK cells were co-cultured with uninfected or HCMV-infected autologous decidual fibroblasts in complete medium in 96 microtiter plate. Controls experiments were performed using dNK cells, uninfected fibroblasts, HCMV-infected autologous fibroblasts that were cultured alone in the same conditions. Cleared supernatants replicates from 4 different experiments were collected after 24 hours of culture and stored at −80°C. Cytokines, chemokines and growth factors levels were measured using a 42-multiplexed Affymetrix cytokine assay according to the manufacturer protocol (Procarta/Ozyme). The following cytokines and chemokines were analyzed: IL-1α (NM_000575), IL-1RA (NM_000577), IL-1β (NM_000576), IL-2 (NM_000586), IL-2RA (NM_000417), IL-4 (NM_000589), IL-5 (NM_000879), IL-6 (NM_000600), IL-8/CXCL8 (NM_0005843), IL-10 (NM_000572), IL-12 (NM_002187), IL-15 (NM_000585), IL-17A (NM_002190), IL-17F (NM_052872), IL-22 (NM_020525.4), IP10/CXCL10 (NM_001565), Basic-FGF/FGF2 (NM_002006), EGF (NM_005429.2), Eotaxin-3/CCL26 (NM_006072), E-selectin/CD62E (NM_000450), sFas (NM_000043), sFasL (NM_000639), Fractalkine/CX3CL1 (NM_002996), GM-CSF (NM_000758), Granzyme B (NM_004131), GROα/CXCL-1 (NM_001511), sICAM-1 (NM_000201), Leptin (NM_000230), IFN-α2 (NM_000605), IFN-β (NM_002176), IFN-γ (NM_000619), IFN-ω (NM_002177), MCP-1/CCL2 (NM_002982), MIP-1α/CCL3 (NM_002983), MIP-1β/CCL4 (NM_002984), RANTES/CCL5 (NM_002985), SDF-1/CXCL12 (NM_000609), TGF-α (NM_001099691), TRAIL (NM_003810), TNF-α (NM_000594), TNF-β (NM_001159740), VEGF-A (NM_001025366). Measurement and analysis were performed using the BioRad Bio-Plex System (BioRad, France). Data points are expressed as follows: Specific dNK cell cytokine-chemokine secretion =  [Total concentration of dNK cells cultured in the presence of fibroblasts - Fibroblasts secretion].

### Explant organ culture preparation and dNK cell tissue invasion

Trophoblastic villous explants established from first trimester elective termination of pregnancy samples. Tissue was minced (1 to 2 mm) and placed in 24 well tissue culture plates in complete tissue culture media (PromoCell, France). After four hours of culture at 37°C and two changes of culture media, explants were at either left uninfected or infected with HCMV AD169 for two days.

For tissue invasion, trophoblast organ culture nuclei were stained with 4 pM DAPI for 5 min, autologous dNK cells were labeled with 1 µM Cell Tracker Red (Invitrogen) for 15 min. All staining procedures were performed at 37°C and quenched with 10 ml of tissue culture media containing 10% FCS. Each explant (1–2 mm) was incubated with 5×10^5^ dNK cells at 37°C. After two hours of contact, organ explants were gently washed with excess of complete media (4 washes), fixed in 4% paraformaldehyde for 20 min, washed twice in PBS and mounted for two-photon microscopy analysis.

### Two-photon microscopy

Images were taken using Zeiss two-photon microscopy at 900 nm laser excitation. Fluorescence emission was collected using dichroic mirrors to split fluorescence into three channels (blue, green and red). Z stacks were taken at 10 µm slice intervals using Zeiss Zen software. Imaris software was used to analyze the acquired data.

### Immunohistochemistry (IHC) examination

HCMV^+^ whole placental biopsies were obtained from two pathological termination of pregnancy (24.5 weeks and 25 weeks of pregnancy). Tissues were fixed in 10% formalin, embedded in paraffin and processed for IHC as previously described [69] Briefly, 6-µm-thick sections of paraffin-embedded samples were immunostained with an anti-CD56 mAb (1B6 clone) and an anti-HCMV-IE mAb (Argene). Photographs were taken with 40X objective of Leica microscope.

### Statistical analysis

Unpaired Student *t* test was calculated using GraphPad Prism 4.0 (GraphPad Software). Unless otherwise indicated, data represent the mean ± S.D. from at least three independent experiments.

### Gene accession numbers

CD69 (NM_001781), NKp30/NCR3 (NM_001145466), NKp44/NCR2 (NM_001199509), NKp46/NCR1 (NM_001145457), NKG2D/KLRK1 (NM_007360), KIR2DL1/CD158A (NM_014218), KIR2DL2/CD158B1 (NM_014219), KIR2DL3/CD158B2 (NM_015868), KIR2DL4/CD158D (NM_001080770), ILT2/LILRB1/CD85j (NM_001081637), NKG2C/KLRC2 (NM_002260), HLA-A,-B,-C (NM_001242758, NM_005514, NM_001243042), HLA-E (NM_005516), HLA-DR (NM_002124).

## Supporting Information

Figure S1 **Characterization and HCMV-AD169 infectivity of decidual fibroblasts.** (A) Decidual fibroblasts were purified as described in M&M. The purity was analyzed using anti-vimentin (green, fibroblasts) and anti-cytokeratin-7 (red, cytotrophoblast) staining. Nuclei were stained with dapi (cyan). (B) Fibroblasts were infected with HCMV (AD169) for 48 h. Nuclei are stained with dapi (cyan) and HCMV-IE (red). Fibroblasts were stained for vimentin (green), α-tubulin (blue). Bar represent 20 µm. (C) Kinetics of fibroblasts infection was quantified over three days.
(TIF)

Figure S2 **Specificity of effector cell cytotoxicity.** (A) dNK cell cytotoxicity was analyzed against uninfected or AD169-infected autologous decidual fibroblasts after 4 h of contact. (B & C) pNK cell cytotoxicity was analyzed against autologous decidual fibroblasts after 4 h (B) or 18 h (C) assay, mean specific lysis is calculated from triplicates within the same experiment out of four. (D & E) dNK cell cytotoxicity against heterologous decidual fibroblasts analyzed after 4 h (D) or 18 h (E) of contact. Data on the graphs are from one representative experiment out of three. (F) dNK and pNK cell cytotoxicity against K562 classical target cell line after 4 h of contact. (G) dNK cell cytotoxicity towards semi-allogeneic trophoblasts was evaluated in three different decidual samples (Tropho_1, _2 and _3) and compared to lysis of autologous infected decidual fibroblasts. (H) Recombinant FasL and TRAIL induce lysis of Jurkat cell line. Jurkat cells were incubated with recombinant TRAIL (rTRAIL) or FasL (rFasL). Specific lysis was performed in the absence or the presence of blocking antibodies against TRAIL (α-TRAIL) or FasL (α-FasL).
(TIF)

Figure S3 **MTOC polarization and Golgi relocalization to the immune synapse.** Uninfected (AD169^−^) or HCMV-infected (AD169^+^) decidual fibroblasts (F) plated on glass coverslips were incubated with autologous dNK cells (dNK) for 20 min at 37°C. (A) Formed conjugates were fixed and permeabilized for intracellular staining of F-actin (blue), α-tubulin microtubules (green) and Golgin (red) simultaneously. Scale bar represent 20 µm. Enlargement of the synaptic area of conjugates presented in the right panels. Asterisks indicate the MTOC. Arrowheads point to the Golgi apparatus. Scale bar represent 5 µm. (B) Bar graphs show the frequency of conjugates formation between dNK cells and autologous fibroblasts that were either kept uninfected (AD169^−^) or HCMV-infected (AD169^+^). More than 500 fibroblasts (white graphs) and at least 50 conjugates (black graphs) were scored in each experiment (n = 5). Statistical analysis was performed using unpaired Student's *t*-test. ***, *p*<0.001. (C) Immunostaining for F-actin (phalloidin in green), HCMV-IE1 (pink), CD2 (red). Scale bar, 20 µm.
(TIF)

Figure S4 **Analysis of NK cell receptor repertoire during HCMV infection.** (A) dNK cells were co-cultured with autologous fibroblasts that were either kept uninfected or infected with HCMV AD169 for 48 h. dNK cells were stained for surface expression of the indicated receptor using fluorochrome-conjugated antibodies and analyzed by FACS. Representative FACS histograms gated on CD56^pos^ CD3^neg^ dNK cells are shown (n = 5). Specific receptors are indicated below each panel. dNK cells in contact with uninfected fibroblasts are represented by black line, dNK cells in contact with HCMV-infected fibroblasts are represented by shaded gray. Dotted gray line represents isotype-matched control Ig. (B) dNK cells were co-cultured with autologous fibroblasts that were either uninfected or infected with HCMV-AD169 for 18 h. dNK cells were stained for surface expression of the indicated receptor using fluorochrome-conjugated antibodies and analyzed by flow cytometry as indicated above. Representative FACS histograms gated on CD56^pos^ CD3^neg^ dNK cells are shown (n = 5). dNK cells in contact with uninfected fibroblasts are represented by black line, dNK cells in contact with HCMV-infected fibroblasts are represented by shaded gray. Dotted gray line represents isotype-matched control Ig. One representative histogram out of five independent experiments is shown. (C) pNK cells were co-cultured with autologous decidual fibroblasts that were either uninfected or infected with HCMV-VHLE for 18 h. pNK cells were stained for surface expression of the indicated receptor using fluorochrome-conjugated antibodies and analyzed by flow cytometry as indicated above. Representative FACS histograms gated on CD56^pos^ CD3^neg^ pNK cells are shown (n = 3). Cells in contact with uninfected fibroblasts are represented by black line, with VHLE-infected fibroblasts are represented by shaded dark gray. Light gray histograms represent isotype-matched control Ig. One representative histogram out of three independent experiments is shown.
(TIF)

Figure S5 **HCMV infection down-modulates cell surface expression of HLA-E without affecting the total amounts of HLA-E.** (A) HCMV-VHLE infection modulates the expression of NKR ligands on decidual fibroblasts. The binding of human NKp30-Fc, NKp46-Fc, NKp44-Fc, NKG2D-Fc and CD99-Fc chimera was used to evaluate the cell surface expression of specific receptor ligands. HLA-E cell surface expression evaluated using MEM-E/08 mAb. One representative FACS histogram out of three independent experiments is shown. Uninfected (black line), VHLE-infected (shaded dark gray). (B) HLA-E expression evaluated in additional three decidual fibroblasts from three independent deciduas (dFibro_1, _2, _3) or in MRC-5 cell line. HLA-A,-B,-C expression by MRC-5 cells was analyzed by specific mAb. For MRC-5 cells, FACS histograms are representative of three independent experiments. Uninfected (black line), HCMV-infected (shaded dark gray). Light gray (line or shaded) histogram represent isotype-matched control Ig. (C) HLA-E detected by western blot in MRC-5 cells or decidual fibroblast from three different deciduas. Cells were HCMV-infected for 48 h. HLA-E detected by MEM-E/06 (top gel) and anti-β-actin (bottom gel). The size of protein ladder is given in kDa.
(TIF)

Figure S6 **HCMV infection regulates NKR ligand expression in decidual fibroblasts: Role on pNK cell cytotoxicity.** (A) K562 cell line were incubated with CD99-Fc (CTRL), NKp46-Fc, NKp30-Fc chimera and used as target cells to evaluate pNK cell cytotoxicity in a 4 h chromium release assay. (B–E) pNK cells cytotoxicity against uninfected (gray plots) or VHLE-infected autologous decidual fibroblasts (black plots) after 18 h of contact. (B) NKp30-Fc, (C) NKp46-Fc, (D) NKp44-Fc, (E) NKG2D-Fc chimeric receptors were used to block the corresponding specific ligands. CD99-Fc soluble receptor was used as control (CTRL). (F) Analysis of NKG2A and NKG2C/E function was performed in the presence of blocking antibody against HLA-E molecules (α-HLA-E) or isotype matched control. Data sets represent mean lysis ± S.D. from three independent experiments done in replicate. Statistical comparisons were performed using two-way ANOVA test. ***, *p*<0.001.
(TIF)

Figure S7 **HCMV infection modulates Fibroblasts cytokine/chemokine production.** Decidual fibroblasts were kept uninfected (AD169^−^, gray) or AD169-infected (AD169^+^, black) for 48 h with HCMV-AD169 strain. Cytokines were quantified in the supernatants using a 42-multi-plexed cytokine assay. Representative histograms from specific cytokines-chemokines that are produced by uninfected and HCMV-infected decidual fibroblasts are presented. Normalized data points are given as mean values ± S.D. calculated from four independent experiments.
(TIF)

Text S1 **Supplementary material and methods.**
(DOCX)

Video S1 **dNK cells are localized at the external boarder of uninfected autologous trophoblasts.** Three-dimensional reconstruction of dNK cell infiltration of chorionic *villi* organ explant shown in Figure 6. Volume rendering reconstruction and animation were obtained from two-photon Z-stack taken at 10 µm slice intervals using Imaris software of 200 µm section. dNK cells (Cell tracker Red), dapi staining of explants' nuclei (cyan). Images are at 5 frames/s; Scale bar: 100 µm.
(AVI)

Video S2 **dNK cells infiltrate and form “immune synapse-like structures” with AD-169 infected autologous trophoblasts.** Three-dimensional reconstruction of dNK cell infiltrating HCMV-infected chorionic *villi* organ explant shown in Figure 6. Volume rendering reconstruction and animation were obtained as in video S1. Images are at 5 frames/s; Scale bar: 100 µm.
(AVI)
