# Correction: Human Cytomegalovirus Infection Elicits New Decidual Natural Killer Cell Effector Functions

**DOI:** 10.1371/annotation/94476def-de2e-4a6e-b0c2-2f2d2ccae784

**Published:** 2013-05-07

**Authors:** Johan Siewiera, Hicham El Costa, Julie Tabiasco, Alain Berrebi, Géraldine Cartron, Philippe Le Bouteiller, Nabila Jabrane-Ferrat

The sixth author's name was spelled incorrectly. The correct name is: Philippe Le Bouteiller.

