# Supplementary figures and images for: Human Cytomegalovirus Infection Elicits New Decidual Natural Killer Cell Effector Functions
Source: PLoS Pathog. 2013 Apr 4;9(4):e1003257. doi: 10.1371/journal.ppat.1003257 (PMC3617138; doi:10.1371/journal.ppat.1003257)

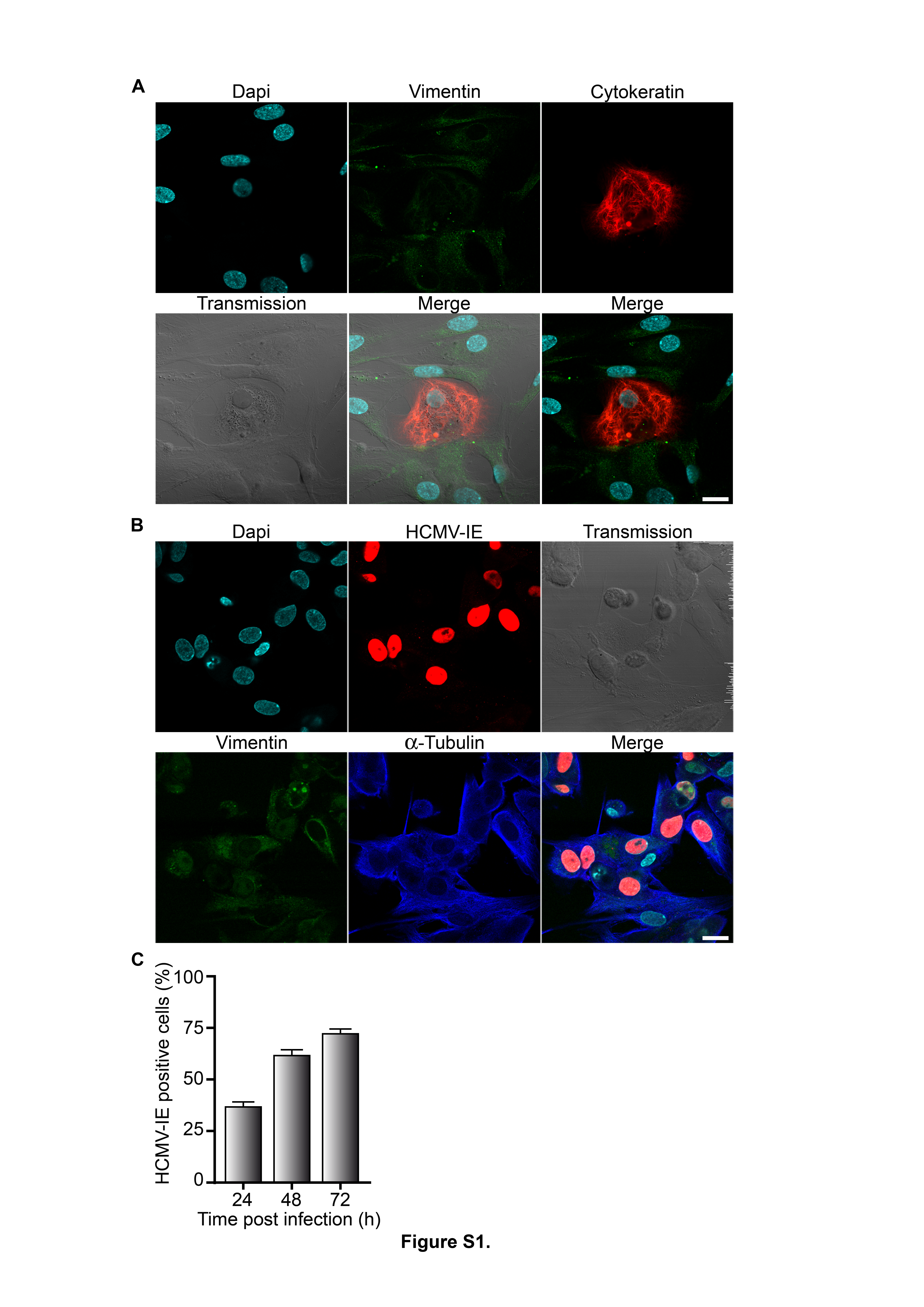

Supplement: Figure S1 — Characterization and HCMV-AD169 infectivity of decidual fibroblasts. (A) Decidual fibroblasts were purified as described in M&M. The purity was analyzed using anti-vimentin (green, fibroblasts) and anti-cytokeratin-7 (red, cytotrophoblast) staining. Nuclei were stained with dapi (cyan). (B) Fibroblasts were infected with HCMV (AD169) for 48 h. Nuclei are stained with dapi (cyan) and HCMV-IE (red). Fibroblasts were stained for vimentin (green), α-tubulin (blue). Bar represent 20 µm. (C) Kinetics of fibroblasts infection was quantified over three days. (TIF) [file ppat.1003257.s001.tif]

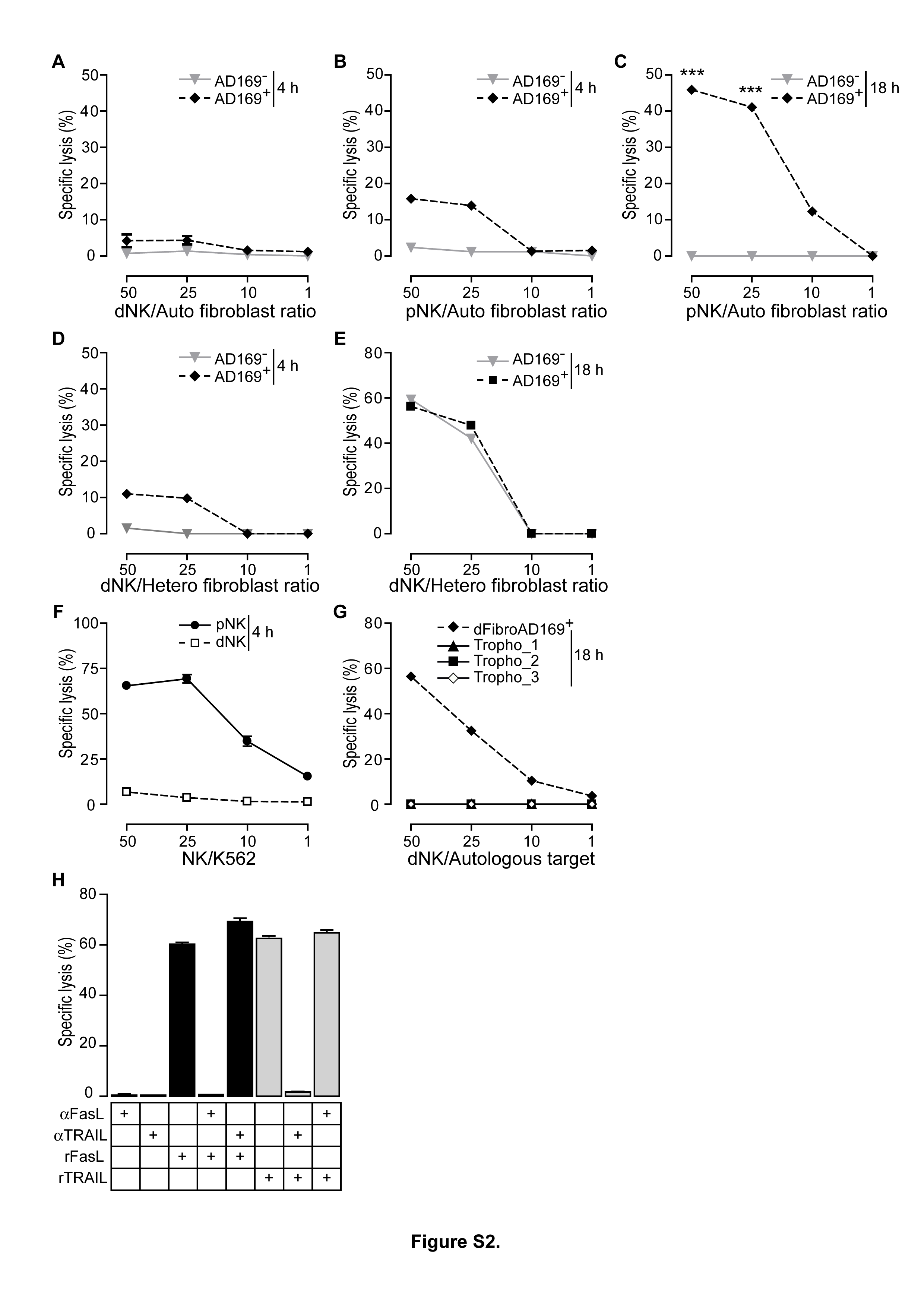

Supplement: Figure S2 — Specificity of effector cell cytotoxicity. (A) dNK cell cytotoxicity was analyzed against uninfected or AD169-infected autologous decidual fibroblasts after 4 h of contact. (B & C) pNK cell cytotoxicity was analyzed against autologous decidual fibroblasts after 4 h (B) or 18 h (C) assay, mean specific lysis is calculated from triplicates within the same experiment out of four. (D & E) dNK cell cytotoxicity against heterologous decidual fibroblasts analyzed after 4 h (D) or 18 h (E) of contact. Data on the graphs are from one representative experiment out of three. (F) dNK and pNK cell cytotoxicity against K562 classical target cell line after 4 h of contact. (G) dNK cell cytotoxicity towards semi-allogeneic trophoblasts was evaluated in three different decidual samples (Tropho_1, _2 and _3) and compared to lysis of autologous infected decidual fibroblasts. (H) Recombinant FasL and TRAIL induce lysis of Jurkat cell line. Jurkat cells were incubated with recombinant TRAIL (rTRAIL) or FasL (rFasL). Specific lysis was performed in the absence or the presence of blocking antibodies against TRAIL (α-TRAIL) or FasL (α-FasL). (TIF) [file ppat.1003257.s002.tif]

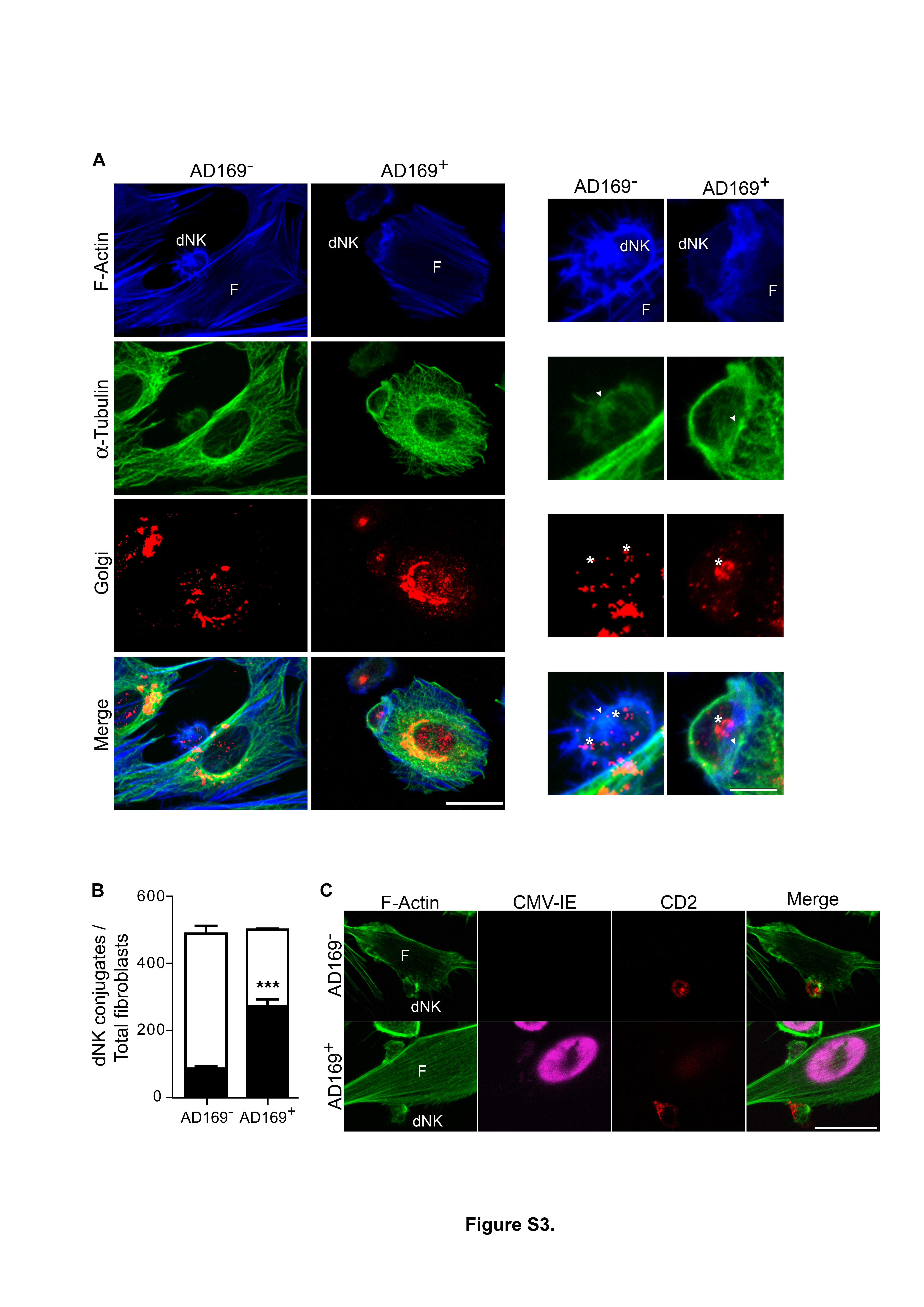

Supplement: Figure S3 — MTOC polarization and Golgi relocalization to the immune synapse. Uninfected (AD169−) or HCMV-infected (AD169+) decidual fibroblasts (F) plated on glass coverslips were incubated with autologous dNK cells (dNK) for 20 min at 37°C. (A) Formed conjugates were fixed and permeabilized for intracellular staining of F-actin (blue), α-tubulin microtubules (green) and Golgin (red) simultaneously. Scale bar represent 20 µm. Enlargement of the synaptic area of conjugates presented in the right panels. Asterisks indicate the MTOC. Arrowheads point to the Golgi apparatus. Scale bar represent 5 µm. (B) Bar graphs show the frequency of conjugates formation between dNK cells and autologous fibroblasts that were either kept uninfected (AD169−) or HCMV-infected (AD169+). More than 500 fibroblasts (white graphs) and at least 50 conjugates (black graphs) were scored in each experiment (n = 5). Statistical analysis was performed using unpaired Student's t-test. ***, p<0.001. (C) Immunostaining for F-actin (phalloidin in green), HCMV-IE1 (pink), CD2 (red). Scale bar, 20 µm. (TIF) [file ppat.1003257.s003.tif]

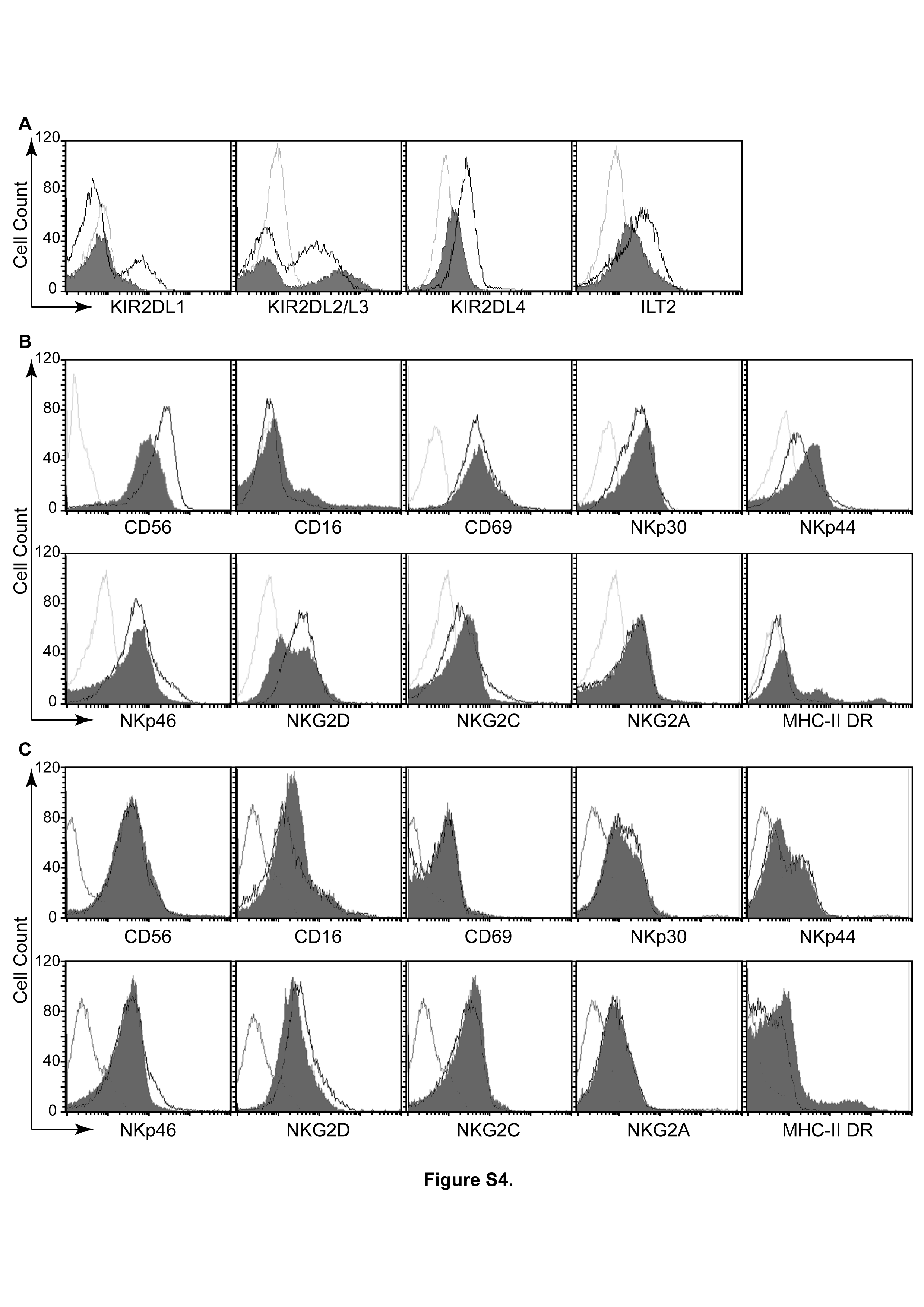

Supplement: Figure S4 — Analysis of NK cell receptor repertoire during HCMV infection. (A) dNK cells were co-cultured with autologous fibroblasts that were either kept uninfected or infected with HCMV AD169 for 48 h. dNK cells were stained for surface expression of the indicated receptor using fluorochrome-conjugated antibodies and analyzed by FACS. Representative FACS histograms gated on CD56pos CD3neg dNK cells are shown (n = 5). Specific receptors are indicated below each panel. dNK cells in contact with uninfected fibroblasts are represented by black line, dNK cells in contact with HCMV-infected fibroblasts are represented by shaded gray. Dotted gray line represents isotype-matched control Ig. (B) dNK cells were co-cultured with autologous fibroblasts that were either uninfected or infected with HCMV-AD169 for 18 h. dNK cells were stained for surface expression of the indicated receptor using fluorochrome-conjugated antibodies and analyzed by flow cytometry as indicated above. Representative FACS histograms gated on CD56pos CD3neg dNK cells are shown (n = 5). dNK cells in contact with uninfected fibroblasts are represented by black line, dNK cells in contact with HCMV-infected fibroblasts are represented by shaded gray. Dotted gray line represents isotype-matched control Ig. One representative histogram out of five independent experiments is shown. (C) pNK cells were co-cultured with autologous decidual fibroblasts that were either uninfected or infected with HCMV-VHLE for 18 h. pNK cells were stained for surface expression of the indicated receptor using fluorochrome-conjugated antibodies and analyzed by flow cytometry as indicated above. Representative FACS histograms gated on CD56pos CD3neg pNK cells are shown (n = 3). Cells in contact with uninfected fibroblasts are represented by black line, with VHLE-infected fibroblasts are represented by shaded dark gray. Light gray histograms represent isotype-matched control Ig. One representative histogram out of three independent experiment [file ppat.1003257.s004.tif]

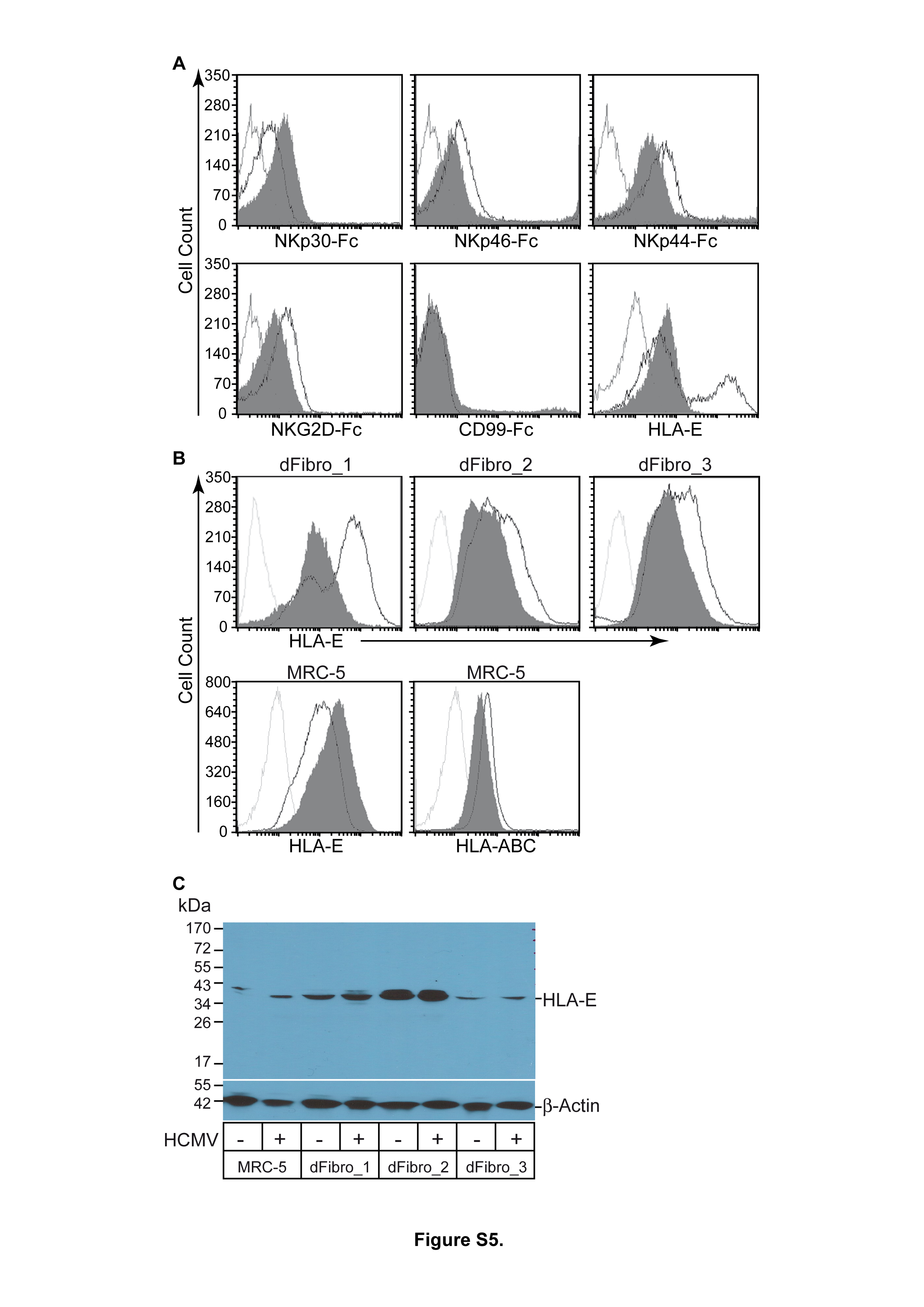

Supplement: Figure S5 — HCMV infection down-modulates cell surface expression of HLA-E without affecting the total amounts of HLA-E. (A) HCMV-VHLE infection modulates the expression of NKR ligands on decidual fibroblasts. The binding of human NKp30-Fc, NKp46-Fc, NKp44-Fc, NKG2D-Fc and CD99-Fc chimera was used to evaluate the cell surface expression of specific receptor ligands. HLA-E cell surface expression evaluated using MEM-E/08 mAb. One representative FACS histogram out of three independent experiments is shown. Uninfected (black line), VHLE-infected (shaded dark gray). (B) HLA-E expression evaluated in additional three decidual fibroblasts from three independent deciduas (dFibro_1, _2, _3) or in MRC-5 cell line. HLA-A,-B,-C expression by MRC-5 cells was analyzed by specific mAb. For MRC-5 cells, FACS histograms are representative of three independent experiments. Uninfected (black line), HCMV-infected (shaded dark gray). Light gray (line or shaded) histogram represent isotype-matched control Ig. (C) HLA-E detected by western blot in MRC-5 cells or decidual fibroblast from three different deciduas. Cells were HCMV-infected for 48 h. HLA-E detected by MEM-E/06 (top gel) and anti-β-actin (bottom gel). The size of protein ladder is given in kDa. (TIF) [file ppat.1003257.s005.tif]

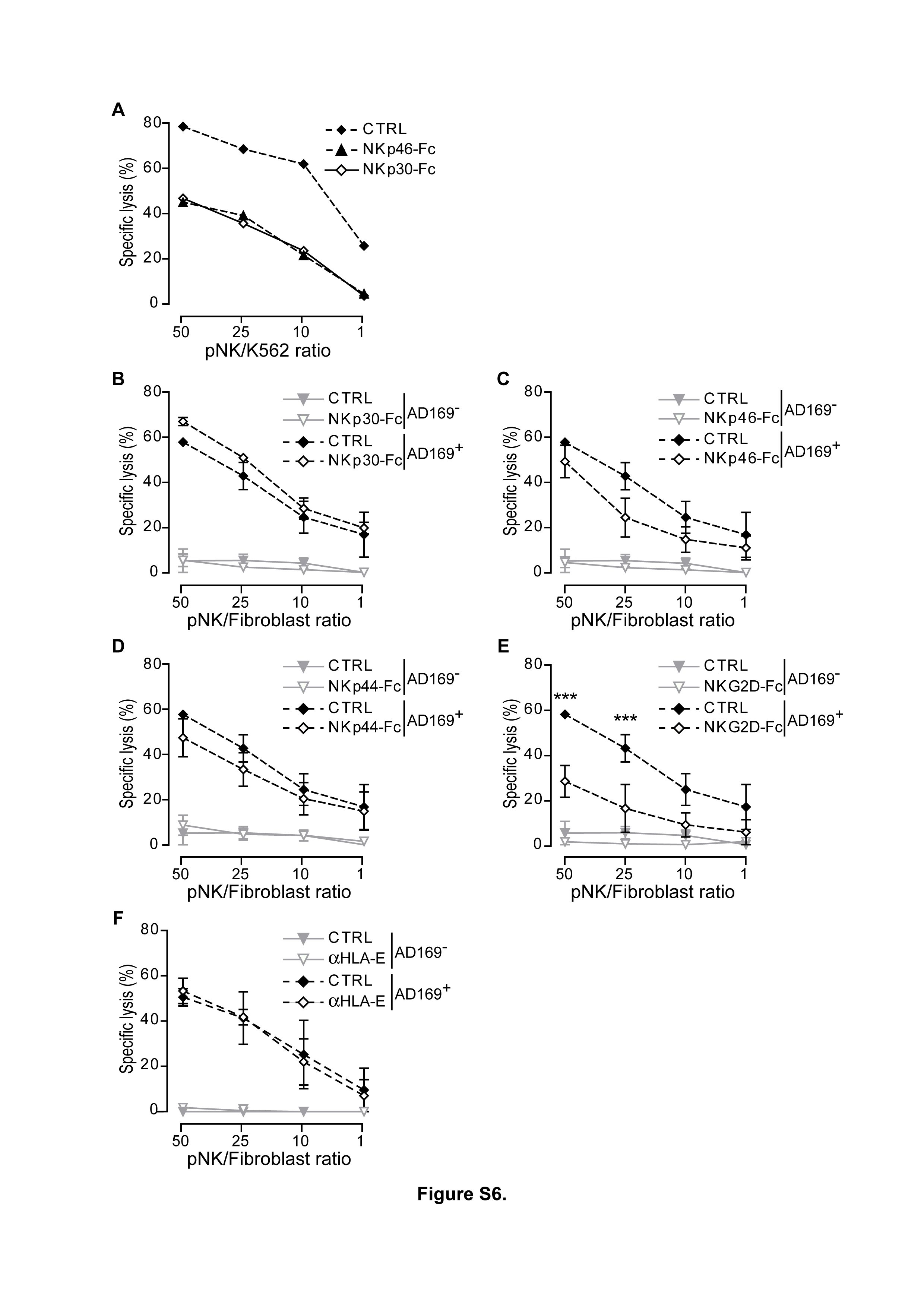

Supplement: Figure S6 — HCMV infection regulates NKR ligand expression in decidual fibroblasts: Role on pNK cell cytotoxicity. (A) K562 cell line were incubated with CD99-Fc (CTRL), NKp46-Fc, NKp30-Fc chimera and used as target cells to evaluate pNK cell cytotoxicity in a 4 h chromium release assay. (B–E) pNK cells cytotoxicity against uninfected (gray plots) or VHLE-infected autologous decidual fibroblasts (black plots) after 18 h of contact. (B) NKp30-Fc, (C) NKp46-Fc, (D) NKp44-Fc, (E) NKG2D-Fc chimeric receptors were used to block the corresponding specific ligands. CD99-Fc soluble receptor was used as control (CTRL). (F) Analysis of NKG2A and NKG2C/E function was performed in the presence of blocking antibody against HLA-E molecules (α-HLA-E) or isotype matched control. Data sets represent mean lysis ± S.D. from three independent experiments done in replicate. Statistical comparisons were performed using two-way ANOVA test. ***, p<0.001. (TIF) [file ppat.1003257.s006.tif]

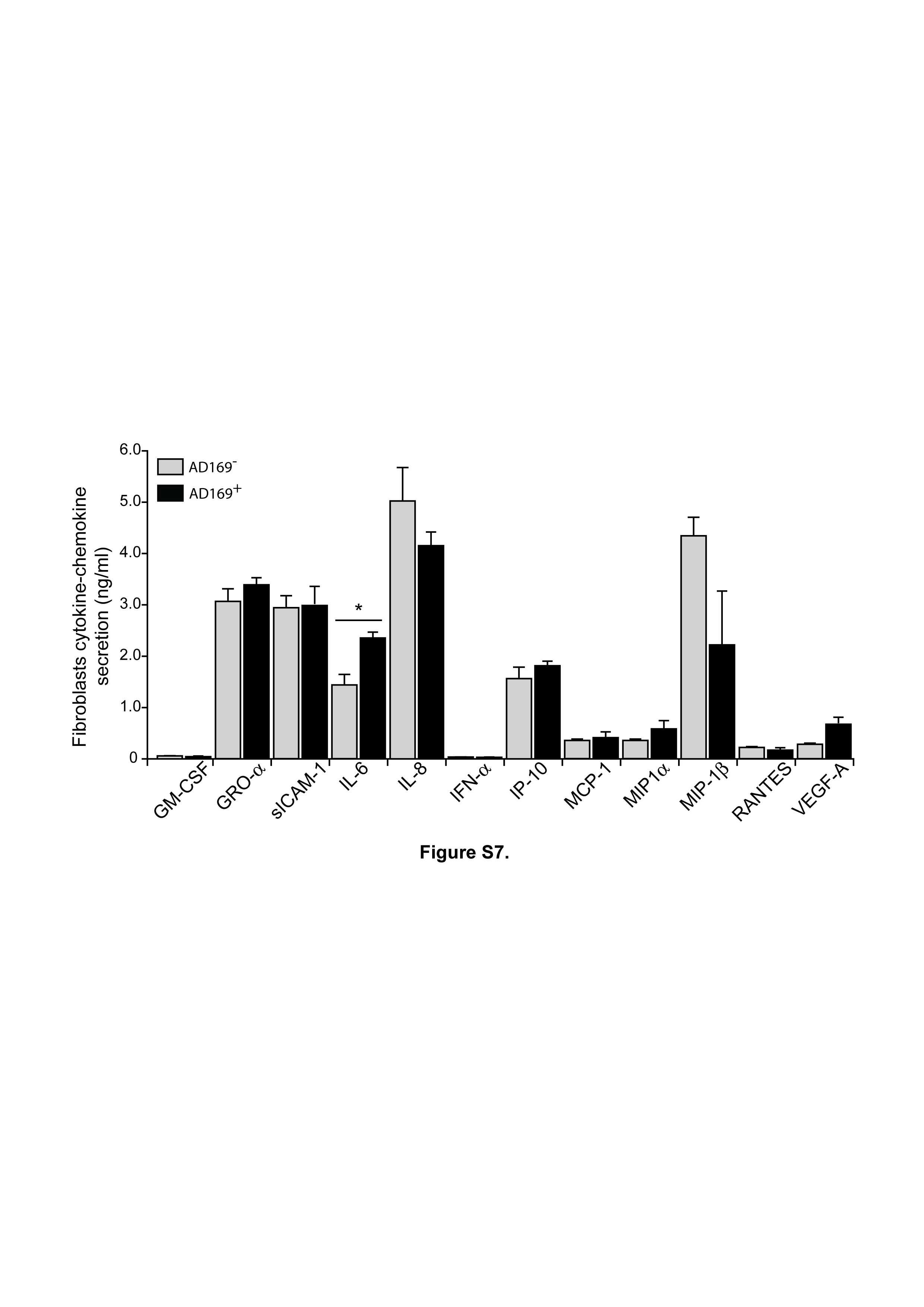

Supplement: Figure S7 — HCMV infection modulates Fibroblasts cytokine/chemokine production. Decidual fibroblasts were kept uninfected (AD169−, gray) or AD169-infected (AD169+, black) for 48 h with HCMV-AD169 strain. Cytokines were quantified in the supernatants using a 42-multi-plexed cytokine assay. Representative histograms from specific cytokines-chemokines that are produced by uninfected and HCMV-infected decidual fibroblasts are presented. Normalized data points are given as mean values ± S.D. calculated from four independent experiments. (TIF) [file ppat.1003257.s007.tif]
