# Supplementary material for: Human Cytomegalovirus Infection Elicits New Decidual Natural Killer Cell Effector Functions
Source: PLoS Pathog. 2013 Apr 4;9(4):e1003257. doi: 10.1371/journal.ppat.1003257 (PMC3617138; doi:10.1371/journal.ppat.1003257)
Supplement: Text S1 — Supplementary material and methods. (DOCX) [file ppat.1003257.s008.docx]

### Supplementary material and methods

**Cell lines**

The following cell lines were used: MRC-5 human fetal lung fibroblast (*ATCC/CCL-171*), and two human lymphoblastic cell lines Jurkat (*ATCC/CRL-1990*) and K562 (*ATCC/****CCL-243*).** Cells were maintained in DMEM (MRC-5) or RPMI (Jurkat and K562) tissue culture media (GIBCO) supplemented with 10% FCS and grown in humidified incubators maintained at 37°C and 5% CO_2_.

**Protein Extracts and Immunoblotting**

Cells were grown in 100-mm dishes were scraped and washed twice in PBS. Total cell lysates were prepared from control and HCMV infected cells [68]. Briefly, cell pellet was resuspended in lysis buffer (50 mM HEPES, pH 7.4, 150 mM NaCl, 1% NP-40, 0.5% sodium deoxycholate, 0.1% SDS) containing 0.1 mg/ml phosphatase and protease inhibitors (Sigma) and incubated on ice for 30 min with repeated mixing. Cleared supernatant containing detergent-soluble proteins were collected. Proteins were quantified with BCA assay kit (Bio-Rad Laboratories, Hercules, CA). Equivalent amounts of proteins from both control and VHLE-infected cells were then resolved by 10% SDS-PAGE and analyzed by western blotting with specific antibodies for HLA-E (MEM-E/06, Exbio) or β-actin (Sigma).
